# Plasmodesmata mediate cell-to-cell transport of brassinosteroid hormones

**DOI:** 10.1038/s41589-023-01346-x

**Published:** 2023-06-26

**Authors:** Yaowei Wang, Jessica Perez-Sancho, Matthieu Pierre Platre, Brenda Callebaut, Marija Smokvarska, Karoll Ferrer, Yongming Luo, Trevor M. Nolan, Takeo Sato, Wolfgang Busch, Philip N. Benfey, Miroslav Kvasnica, Johan M. Winne, Emmanuelle M. Bayer, Nemanja Vukašinović, Eugenia Russinova

**Affiliations:** 1 Department of Plant Biotechnology and Bioinformatics, Ghent University, 9052 Ghent, Belgium; 2 Center for Plant Systems Biology, VIB, 9052 Ghent, Belgium; 3 Laboratoire de Biogenèse Membranaire, Unité Mixte de Recherche 5200, Université de Bordeaux, Centre National de la Recherche Scientifique, 33140 Villenave d’Ornon, France; 4 Plant Molecular and Cellular Biology Laboratory and Integrative Biology Laboratory, Salk Institute for Biological Studies, La Jolla, CA 92037, USA; 5 Department of Organic and Macromolecular Chemistry, Ghent University, 9000 Ghent, Belgium; 6 Laboratory of Growth Regulators, Institute of Experimental Botany, The Czech Academy of Sciences and Palacky Universitý, 77900 Olomouc, Czech Republic; 7 Faculty of Science, Hokkaido University, Sapporo, Japan; 8 Department of Biology, Duke University; Durham, USA; 9 Howard Hughes Medical Institute, Duke University; Durham, USA

## Abstract

Brassinosteroids (BRs) are steroidal phytohormones that are essential for plant growth, development, and adaptation to environmental stresses. BRs act in a dose-dependent manner and they do not travel over long distances, hence, BR homeostasis maintenance is critical for their function. Biosynthesis of bioactive BRs relies on cell-to-cell movement of hormone precursors. However, the mechanism of the short-distance BR transport is unknown and its contribution to control of endogenous BR levels remains unexplored. Here, we demonstrate that plasmodesmata (PD) mediate the passage of BRs between neighboring cells. Intracellular BRs content, in turn, is capable of modulating PD permeability to optimize its own mobility, thereby manipulating BR biosynthesis and signaling. Our work uncovers a thus far unknown mode of steroid transport in eukaryotes and exposes an additional layer of BR homeostasis regulation in plants.

## Introduction

Brassinosteroids (BRs) are steroidal phytohormones that are crucial for plant growth and regulate diverse developmental processes such as cell elongation, cell division, photomorphogenesis, xylem differentiation, as well as biotic and abiotic stress responses^1^. BRs are perceived by the plasma membrane-localized receptor kinase BR INSENSITIVE1 (BRI1) and its coreceptor BRI1-ASSOCIATED-KINASE1 (BAK1) in the apoplast, triggering a sequential phosphorylation between BRI1 and BAK1^2^. This step is required for BRI1 activation and leads to downstream phosphorylation and dephosphorylation events that activate the transcription factors, BRASSINAZOLE RESISTANT1 (BZR1) and BR INSENSITIVE EMS SUPPRESSOR1 (BES1)/BZR2, either to induce or to repress genes in the nucleus as the final outcome of the BR response^3^.

Normally, the hormone level at any given site within the plant is determined by the relative rates of biosynthesis, catabolism, and transport^4^. Until now, a negative transcriptional feedback loop has been considered the main regulatory mechanism of BR biosynthesis and catabolism, in which the transcription of BR biosynthetic and catabolic genes is directly regulated by BES1 and BZR1^5^. Nevertheless, in the *Arabidopsis thaliana* root, elongation zone is the site of maximum BR biosynthetic expression^6^, as well as signaling levels, indicating that additional mechanisms control expression of biosynthetic enzymes. In the root meristem, BR biosynthetic enzymes’ expression domains are separated which implies cell-to-cell movement of hormone precursors^6^ that would allow biosynthetic pathway completion. Hence, the question arises if BR homeostasis in the root meristem is also mediated by short-distance BR transport, as well as what is the mode of BR movement between neighboring cells.

BRs are derived from campesterol and converted to the most bioactive BR, brassinolide (BL) after a series of reactions, including reduction, hydroxylation, epimerization, and oxidation^7^. All enzymes involved in BR biosynthesis, except the steroid 5-α-reductase, DE-ETIOLATED 2 (DET2), are members of the cytochrome P450 (CYP) protein superfamily^16^. The CYPs are well conserved and are predominantly localized in the endoplasmic reticulum (ER)^8^ membrane. For two of the BR CYPs, DWARF4 (DWF4) and CYP85A2, an ER localization has already been demonstrated^9,10^. Nonetheless, how BRs exit the cell after their synthesis in the ER membranes and how they are transported between neighboring cells is still unknown. The mammalian steroid hormones, after their release from steroidogenic cells, are transported to the target cells through the blood by their carrier proteins^11^. However, no homologs of the mammalian steroid carrier proteins are found in plants and BRs do not undergo long-distance movement^12^.

Multicellular organisms rely on cell-to-cell communication to coordinate growth and development across tissues and organs^13^. In plants, plasmodesmata (PD) are critical cytoplasmic communication channels^14^. PD cross the cell walls of adjacent cells and enable intercellular movement of nutrients, hormones, RNAs, proteins, metabolites, and viruses^15–19^. The typical structure of PD consists of a cytoplasmic sleeve, a plasma membrane leaflet, and desmotubule, a membranous cortical ER-derived rod, connecting adjacent cells^20^. PD connectivity is exceptionally dynamic, responds to both internal and external stimuli, and is regulated by the deposition of callose, a β-(1,3)-glucan polymer, in the PD neck^20^. Localized production of the polymer by callose synthases can narrow the cytoplasmic sleeve, whereas degradation mediated by β-1,3-glucanases can widen the channel^20^. Nevertheless, PD conductivity is not directly correlated with the size of the cytoplasmic sleeve^21^, and how exactly PD mediate exchange of molecules between neighboring cells remains poorly understood. PD number is also controlled, because for instance, secondary PD are often formed during cell growth either to maintain or to increase the PD density in expanding cell walls^22^. Whether BR precursors are transported via PD to complete the biosynthetic pathway or initiate signaling in adjacent cell files is not known.

Here, we corroborate the subcellular localization of BR biosynthetic enzymes in the ER, where hormone biosynthesis likely occurs. By combining genetic and bioorthogonal chemistry approaches, we demonstrate that PD are involved in the short-distance transport of BR precursors, which is required for biosynthetic pathway completion. Finally, we provide evidence that the intracellular BR content negatively regulates PD permeability, possibly through BR signaling inducing callose deposition in the cell walls near the neck zone of the PD and, ultimately, affecting its own mobility and biosynthesis efficiency. Our work reveals an unidentified transport mode of steroidal hormones in eukaryotes and presents an additional layer of BR biosynthesis regulation in plants.

## Results

### Symplastic communication modulates BR signaling.

**Extended Data Fig. 1 |:**
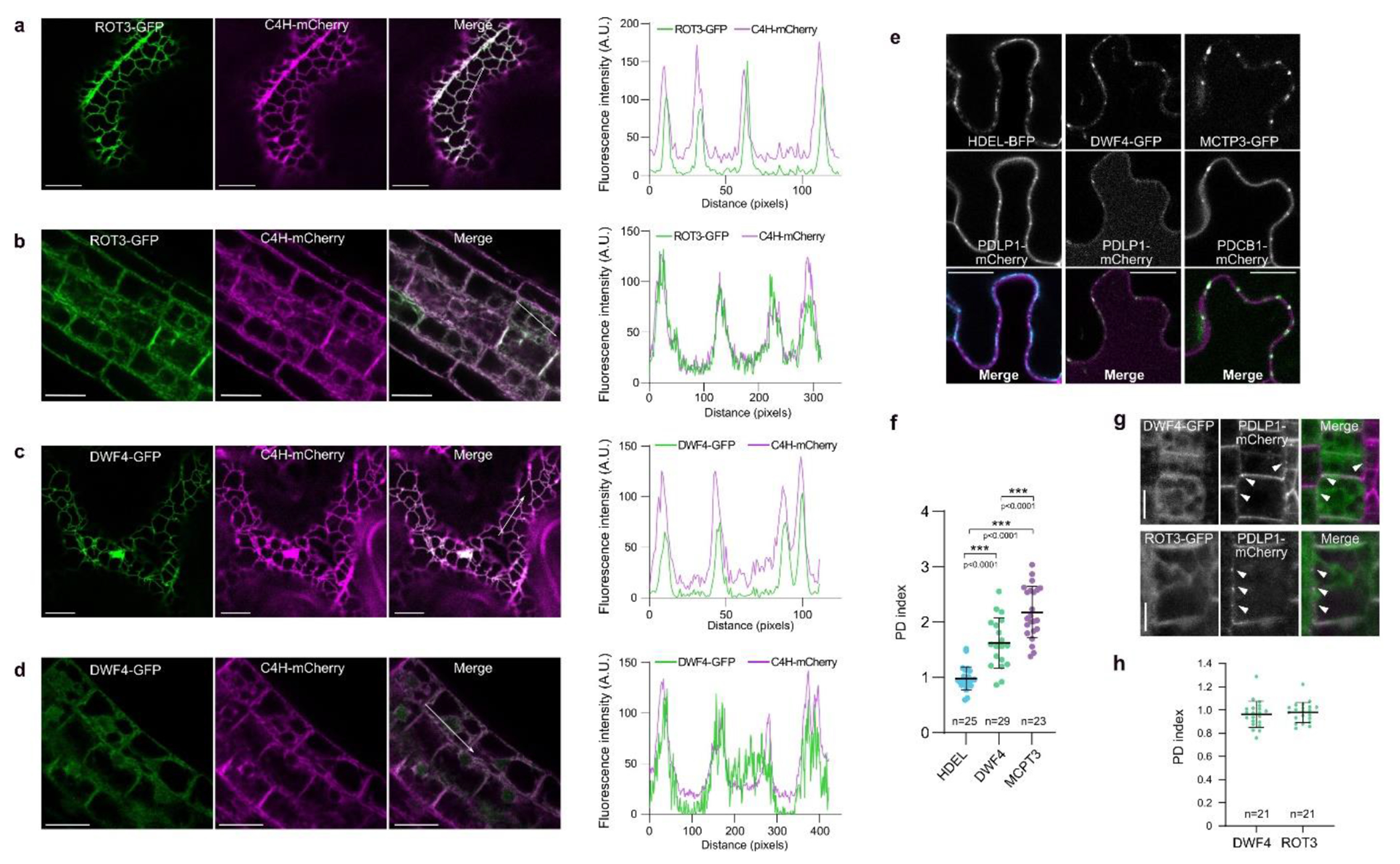
Subcellular localization of BR biosynthetic enzymes. **a,b,c,d**, The BR biosynthetic enzyme ROT3-GFP and DWF4-GFP co-localize with the ER marker C4H-mCherry when transiently co-expressed in tobacco leaves (**a,c**) and in *Arabidopsis* root epidermal cells expressing ROT3-GFP and DWF4-GFP under the control of their native promoters (**b,d**). The fluorescence intensity profiles along the white arrows are shown on the right in (**a,b,c** and **d**). A. U., arbitrary units, **e**, Co-localization of HDEL-BFP and DWF4-GFP with plasmodesmata (PD) marker PDLP1-mCherry and MCTP3-GFP with PD marker PDCB1-mCherry when transiently co-expressed in tobacco leaves. These co-expression combinations were used to calculate PD indexes in (**f**). **f**, The PD index of DWF4-GFP biosynthetic enzyme compared to the indexes of ER marker HDEL-BFP and PD-resident protein MCTP3-GFP. DWF4-GFP index above 1 indicates partial enrichment at PD. All individual data points are plotted. Horizontal and error bars represent the means and s.d., respectively. *n*, number of ROIs used to calculate average PD index. The significant differences were determined with one-way analysis of variance (ANOVA) and Tukey’s multiple comparison tests. *** **P** < 0.001, ** *P* < 0.01, and * *P* < 0.05. **g**, Co-localization of DWF4-GFP and ROT3-GFP biosynthetic enzymes expressed under their native promoters with the PD marker PDLP1-mCherry in *Arabidopsis* roots. Epidermal and cortical cells of the root transition zone were imaged for DWF4-GFP and ROT3-GFP, respectively. White arrowheads mark PD, labeled by PDLP1-mCherry. No clear co-localization of BR biosynthetic enzymes and PDLP1-mCherry were observed. Scale bars, 10 μm ((**a,c,g**), 20 μm (**e**) and 25 μm (**b,d**). For **a,b,c,d,e,f**, the experiment was repeated three times and for **g,h**, twice with similar results.

To determine the subcellular localization of BR biosynthetic enzymes, we co-expressed the GFP-tagged ROTUNDIFOLIA3 (ROT3) and DWF4 with the mCherry-tagged ER membrane-resident marker CINNAMATE 4-HYDROXYLASE (C4H)^23^ in *Nicotiana benthamiana* (tobacco) leaves (Extended Data Fig. 1a,c) and in roots of transgenic *Arabidopsis* plants (Extended Data Fig. 1b,d). As reported earlier for some Arabidopsis CYP proteins^9,10,24^, both enzymes localized in the ER membrane, marking it as the likely site of BR biosynthesis within cells.

**Extended Data Fig. 2 |:**
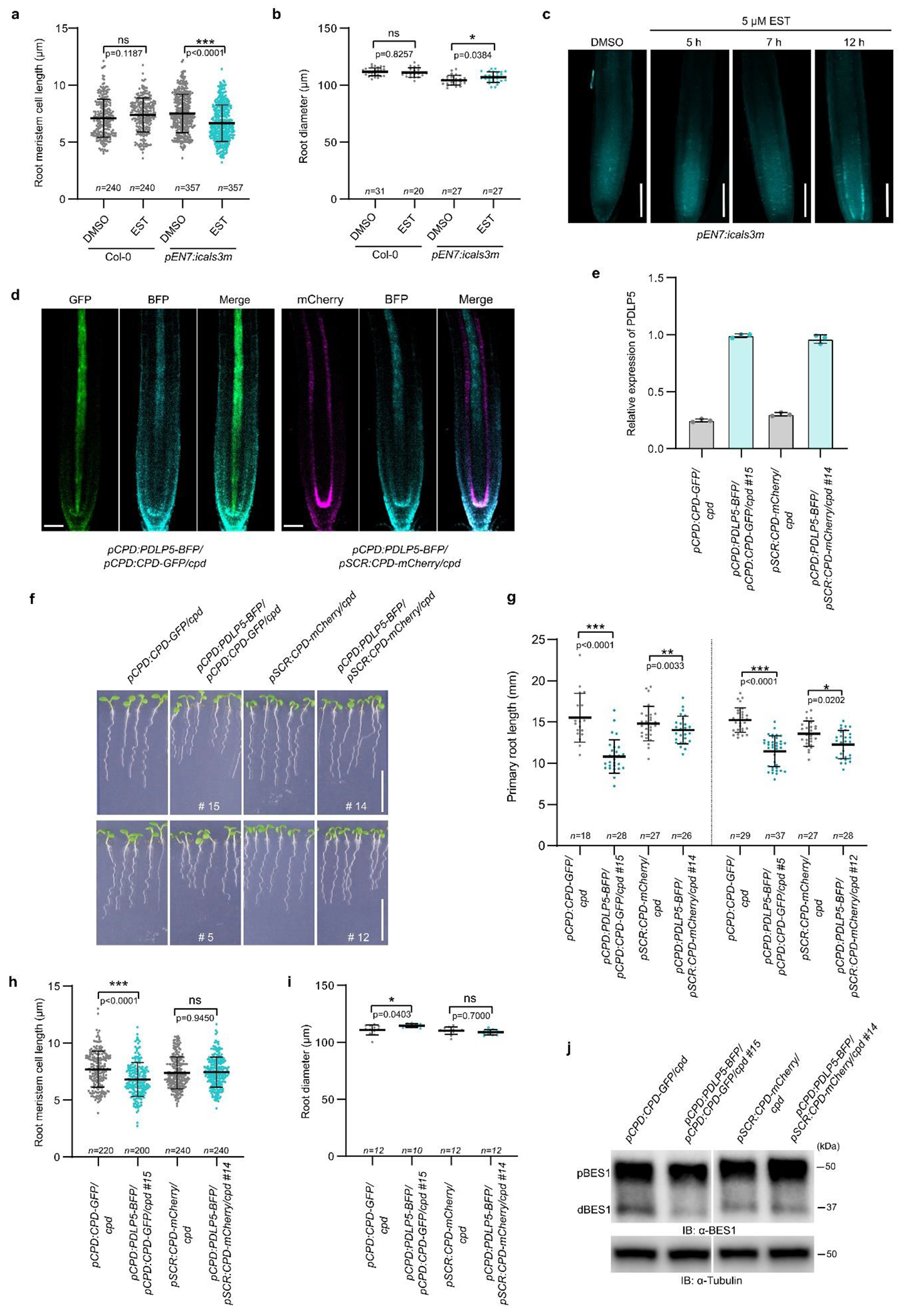
Reduced cell-to-cell connectivity negatively affects BR signaling. **a,b,** Quantification of root meristem cell length in (**a**) and root diameter in (**b**) of five-day-old Col-0 and *pEN7:icals3m* seedlings which were grown for 12 h on agar medium containing either estradiol (EST) (5 μM) or DMSO (mock). **c**, Aniline blue staining of callose deposition in the root tips of *pEN7:icals3m* plants. Five-day-old seedlings were transferred to agar medium containing estradiol (EST) (5 μM) for the indicated times and DMSO (mock), followed by aniline blue staining. Scale bars, 100 μm. **d**, Confocal images of *pCPD:PDLP5-BFP/pCPD:CPD-GFP/cpd* and *pCPD:PDLP5-BFP/pSCR:CPD-mCherry/cpd* lines. Scale bars, 50 μm. **e**, The relative gene expression of *PDLP5* in *pCPD:PDLP5-BFP/pCPD:CPD-GFP/cpd* (line #15) and *pCPD:PDLP5-BFP/pSCR:CPD-mCherry/cpd* (line #14) compared to their segregating siblings that do not express *pCPD:PDLP5-BFP*. Error bars represent s.d. **f**, Phenotypes of 6-day-old *pCPD:CPD-GFP/cpd, pCPD:PDLP5-BFP/pCPD:CPD-GFP/cpd, pSCR:CPD-mCherry/cpd*, and *pCPD:PDLP5-BFP/pSCR:CPD-mCherry/cpd* seedlings from two independent transgenic lines. For each line, segregating siblings that do not express *pCPD:PDLP5-BFP* are shown. Scale bar, 1 cm. **g**, The quantification of primary root length of transgenic lines shown in (**f**). **h,i**, Quantification of root meristem cell length in (**h**) and root diameter in (**i**) of seedlings shown in (**f**). **j**, Phosphorylation status of BES1 detected by immunoblotting (IB) with α-BES1 antibody in roots. Tubulin detected with α-tubulin antibody was used as loading control. pBES1, phosphorylated BES1, dBES1, dephosphorylated BES1. Two panels form each row are from the same blots and were cropped and arranged for clarity. For (**a**,**b**,**g**,**h,i**) all individual data points are plotted. Horizontal and error bars represent the means and s.d., respectively. *n*, number of roots used in (**b,g,i**) and cells used in (**a,h**). The significant differences were determined with one-way analysis of variance (ANOVA) and Tukey’s multiple comparison tests. *** *P* < 0.001, ** *P* < 0.01, and * *P* < 0.05. For **a,b,h,i,j**, the experiment was repeated twice and for **f,g**, three times with similar results.

**Fig. 1 |:**
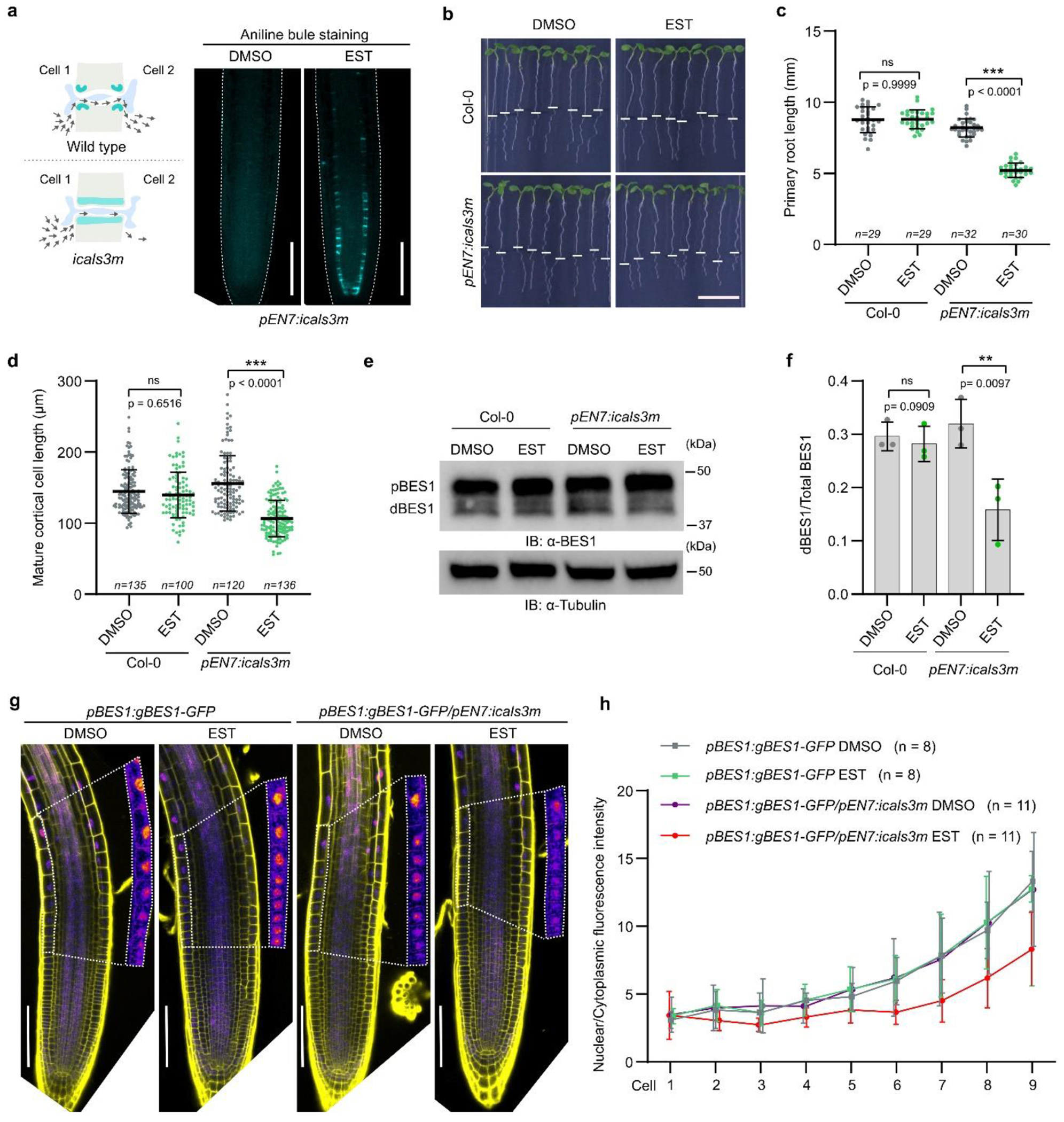
PD permeability modulates BR signaling. **a**, Increased callose biosynthesis at plasmodesmata in **isals3m** expressing plants inhibits exchange of molecules between neighboring cells (left). Aniline blue staining of callose deposition in endodermis of the root tips of *pEN7:icals3m* plants upon estradiol (EST) (5 μM) or DMSO (mock) treatment for 48 h (right). Scale bars, 100 μm. **b**, Induction of callose deposition (48 h EST treatment) in the endodermis of *pEN7:icals3m* seedlings causes root growth defects. Root tips were marked immediately after the transfer (white bars). Scale bar, 1 cm. **c,d**, Quantification of the primary root length (**c**) and mature cortical cell length (**d**) of seedlings shown in (**b**). All individual data points are plotted. Horizontal and error bars represent the means and the represent s.d., respectively. *n*, number of roots used in (**c**) and cells used in (**d**). The significant differences were determined with one-way analysis of variance (ANOVA) and Tukey’s multiple comparison tests. **e**, Phosphorylation status of BES1 detected by immunoblotting (IB) with α-BES1 antibody. Tubulin detected with α-tubulin antibody was used as loading control. Root tips of five-day-old *Arabidopsis* seedlings grown as in (**a**) and induced for 12 h were used. **f**, Quantification of BES1 dephosphorylation in (**e**) presented as a ratio of dephosphorylated BES1 (dBES1) relative to the total BES1. pBES1, phosphorylated BES1. The significant difference was determined with two-tailed Student’s paired *t*-test analysis. Error bars represent s.d. **g**, Confocal images of root tips of *pBES1:gBES1-GFP/Col-0* and *pBES1:gBES1-GFP/pEN7:icals3m* seedlings. Five-day-old seedlings were transferred to agar media containing EST (5 μM) or DMSO (mock) and imaged after 12 h. Cell walls were stained with propidium iodide (PI). Nine cells used for florescence intensity measurements in (**h**) are shown in inset panels (1.5x enlarged). In inset panels, only GFP signal is shown. Brightness and contrast were equally adjusted for each treatment. Scale bars, 100 μm. **h**, Quantification of nuclear/cytoplasmic BES1-GFP fluorescence intensity ratio of epidermal cells in the root transition zone (nine cells shown in inset panels in **g**). Values represent means and error bars represent s.d. *n*, number of roots used for each treatment. *** *p* < 0.001, ** *P* < 0.01, and * *P* < 0.05 for (**c, d, f**). For **b,c,d,e,f,g,h**, experiments were performed in three repeats with similar results.

Previously we showed that BRs move between neighboring cell files in roots of *Arabidopsis thaliana*^6^. Given that BR biosynthetic enzymes are localized in the ER, and the hormone is synthesized intracellularly, we hypothesized that the exchange of BRs between adjacent cell files occurs *via* PD. First, we asked whether the sites of BR biosynthesis might coincide with PD, which could facilitate hormone transport. To this end, we examined colocalization of BR biosynthetic enzyme DWF4-GFP and the mCherry-tagged PD marker PLASMODESMATA-LOCATED PROTEIN1 (PDLP1)^14^ in tobacco leaves (Extended Data Fig. 1e) and estimated the PD enrichment by calculating the PD index^25^ (Extended Data Fig. 1f). DWF4-GFP exhibited a higher PD index than the ER lumen marker HDEL-BFP, indicating a PD association, but these values were lower than those for MCTP3-GFP, a PD resident protein^26^. However, in roots of Arabidopsis plants co-expressing ROT3-GFP and DWF4-GFP with PDLP1-mCherry no clear enrichment of the GFP signal at the PD was found (Extended Data Fig. 1g,h). These findings suggest that BR biosynthetic machinery might be loosely associated with PD and only in certain cell types. Second, we tested if BRs move through the PD. For this, we examined the root growth of transgenic *Arabidopsis* plants expressing the gain-of-function *CALLOSE SYNTHASE3 (CALS3)* from an estradiol-inducible *ENDODERMIS7* (*EN7*) promoter (*pEN7:icals3m*) that can block PD by callose deposition in the PD neck in the endodermis after induction^27^ (Fig. 1a). The phenotypic analysis showed that after a 48 h estradiol treatment primary root growth was arrested, and that the length of the mature cortical cells was significantly shorter than that of the mock-treated plants (Fig. 1b–d). To investigate whether blocking PD in the endodermis affects BR signaling, we analyzed BR-specific phenotypes in the root meristems^6^. To look at relatively early BR signaling responses, and minimize pleiotropic effect of PD closure, we decreased the induction time to 12 h, as this condition was sufficient to observe callose production (Extended Data Fig. 2c). Callose deposition reduced the average length of meristematic cortical cells and slightly increased root diameter (Extended Data Fig. 2a,b), suggesting decreased BR signaling levels in *pEN7:icals3m* roots after induction. We then analyzed the accumulation of dephosphorylated BES1, which can be used as a readout for BR signaling activation^3^. Compared to Col-0, BES1 was slightly less dephosphorylated in the callose-induced roots (Fig. 1e,f), indicating a decrease in BR signaling. In addition, we introduced the *pBES1:gBES1-GFP* reporter^28^ into the *pEN7:icals3m* plants^27^ and examined the nuclear accumulation of BES1, which is also used as an indicator of BR signaling activation. The nuclear/cytoplasm fluorescent intensity ratio of BES1-GFP was reduced in root epidermal cells after 12 h of estradiol treatment (Fig. 1g,h), possibly as a result of decreased BR signaling. These findings suggest that the reduced PD permeability might compromise BR signaling, probably due to impaired transport of BRs through the PD.

Limiting the PD transport can be detrimental for root growth, since numerous signaling components and nutrients are transported through these intercellular channels^15–19^. To overcome, or at least limit the pleiotropic effects of callose-plugged PD on root growth and uncouple BR-specific effects, we ectopically expressed the PD-LOCATED PROTEIN5 (PDLP5)-BFP^29^ fusion to induce overaccumulation of callose at the PD in the native domain of the BR biosynthetic enzyme, CONSTITUTIVE PHOTOMORPHOGENESIS AND DWARFISM (CPD) in the *pCPD:CPD-GFP/cpd* (Extended Data Fig. 2d,e). This allowed us to spatially link CPD enzyme activity with ectopic callose deposition in the central part of the root, the stele, and minimize interruption of movement of other PD-transported molecules. Roots of this transgenic line grew slower compared to *pCPD:CPD-GFP/cpd* ones (Extended Data Fig. 2f,g) and exhibited BR deficient phenotypes (Extended Data Fig. 2h,i). Additionally, an increase in phosphorylated BES1 indicated reduced BR signaling levels in roots expressing PDLP5-BFP (Extended Data Fig. 2j).

To validate our findings that impaired symplastic transport negatively regulates BR signaling, we compared the effect of PDLP5-BFP expression under the *CPD* promoter in *pCPD:CPD-GFP/cpd* and *pSCR:CPD-mCherry/cpd* backgrounds. When the CPD-mCherry^6^ enzyme was ectopically expressed in the endodermis, milder growth defects were observed (Extended Data Fig. 2d–i), as well as less affected BR signaling levels (Extended Data Fig. 2j). CPD is a BR biosynthetic enzyme with the most restricted expression domain in the stele, while all other enzymes are, at least partially, expressed in other tissues^6^. Therefore, displacement of CPD enzyme expression to the endodermis allowed possible movement of BR precursors and completion of the pathway as a result of bypassing the PDLP5-BFP-induced callose deposition and respectively, the reduced PD connectivity in the stele.

**Extended Data Fig. 3|:**
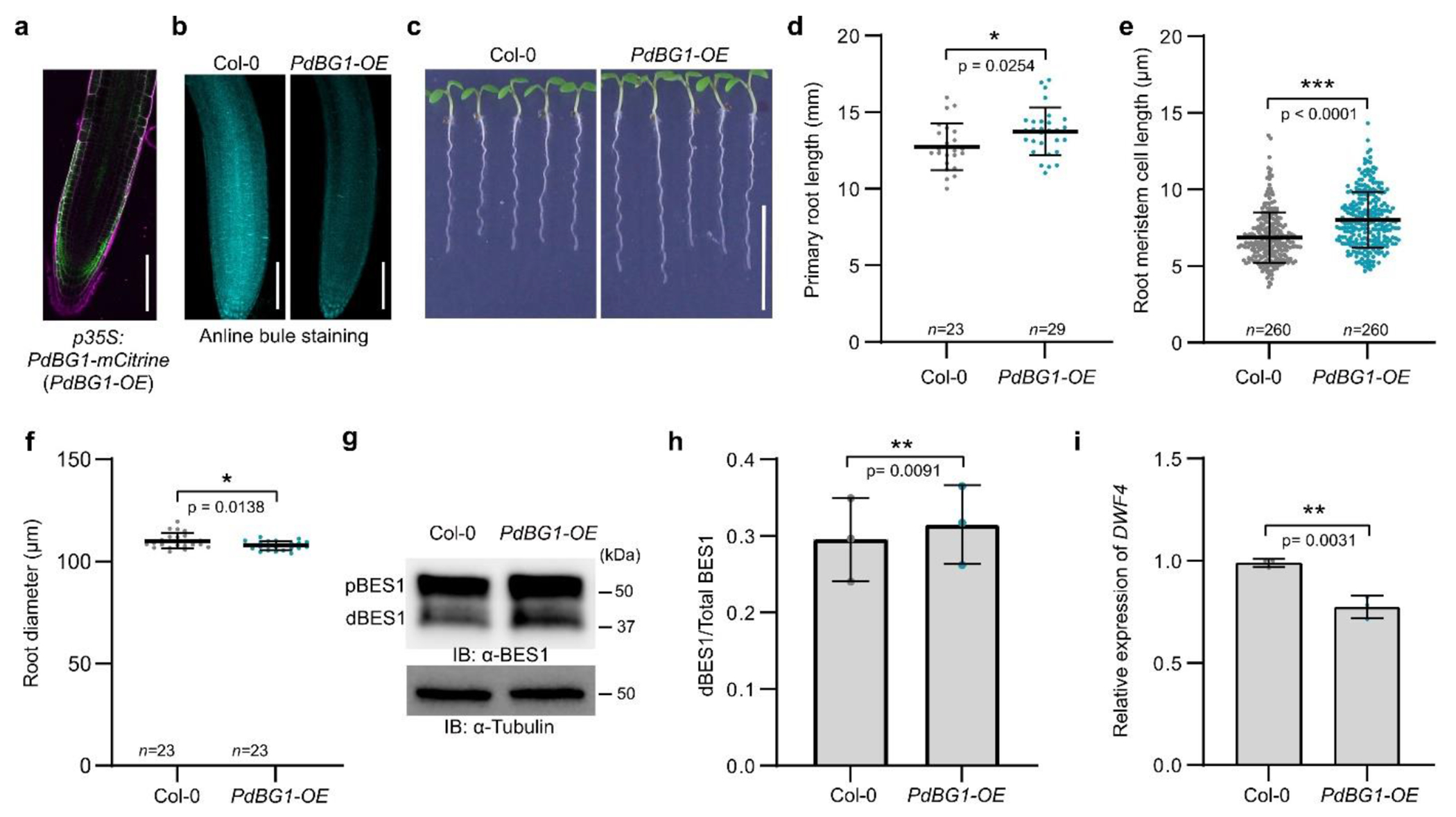
Increased cell-to-cell connectivity positively affects BR signaling. **a**, The confocal image of 6-day-old *p35S:PdBG1-mCitrine* (PdBG1-OE) root meristem. Cell walls were stained with propidium iodide. **b**, Aniline blue staining of callose deposition in the root tips of PdBG1-OE plants. Scale bars, 100 μm (**a,b**). **c**, Phenotype of 6-day-old PdBG1-OE seedlings. Scale bar, 1 cm. **d,e,f**, Quantification of the primary root length in (**d**), root meristem cell length in (**e**) and root meristem diameter in (**f**) shown in (**c**). All individual data points are plotted. Horizontal and error bars represent the means and s.d., respectively. *n*, number of roots used in (*d,f*) and cells used in (*e*). The significant difference was determined with two-tailed Student’s unpaired *t*-test analysis. *** *P* < 0.001, ** *P* < 0.01, and * *P* < 0.05. **g**, Phosphorylation status of BES1 detected by immunoblotting with α-BES1 antibody in whole seedlings. Tubulin detected with α-tubulin antibody was used as loading control. pBES1, phosphorylated BES1, dBES1, dephosphorylated BES1. **h**, Quantification of BES1 dephosphorylation in (**g**) represented as a ratio of dephosphorylated BES1 (dBES1) relative to the total BES1. pBES1, phosphorylated BES1. The significant difference was determined with two-tailed Student’s paired *t*-test analysis. *** *P* < 0.001, ** *P* < 0.01, and * *P* < 0.05. **i**, The relative gene expression of *DWF4* in Col-0 and PdBG1-OE line. Error bars represent the s.d. The significant difference was determined with two-tailed Student’s unpaired *t*-test analysis. *** *P* < 0.001, ** *P* < 0.01, and * *P* < 0.05. For **c,d,e,f**, the experiment was repeated twice and for **g,h,i**, in three independent biological repeats with similar results.

Subsequently, we assessed the BR-related phenotypes and BR signaling in transgenic *Arabidopsis* plants overexpressing the callose-degrading enzyme PLASMODESMAL-LOCALIZED B-1,3-GLUCANASE1 (PdBG1)^30^ (*p35S:PdBG1-mCitrine*/Col-0 PdBG1-OE), which have increased PD permeability as a result of enhanced callose turnover (Extended Data Fig. 3a,b). The phenotypic analysis revealed that the length of the primary roots of PdBG1-OE seedlings were significantly longer than those of wild type, with longer meristematic cells and slightly smaller root radius (Extended Data Fig. 3d–f), thus, resembling to certain extent the phenotype of plants with constitutive BR responses^6^. In agreement, the PdBG1-OE plants accumulated more dephosphorylated BES1 than the control (Extended Data Fig. 3g,h), while the expression of *DWF4* was downregulated (Extended Data Fig. 3i), indicating that the increased cell-to-cell connectivity positively affected BR signaling. Taken together, our results show that PD permeability can moderately modulate BR signaling levels.

### PD mediate BR movement.

**Extended Data Fig. 4|:**
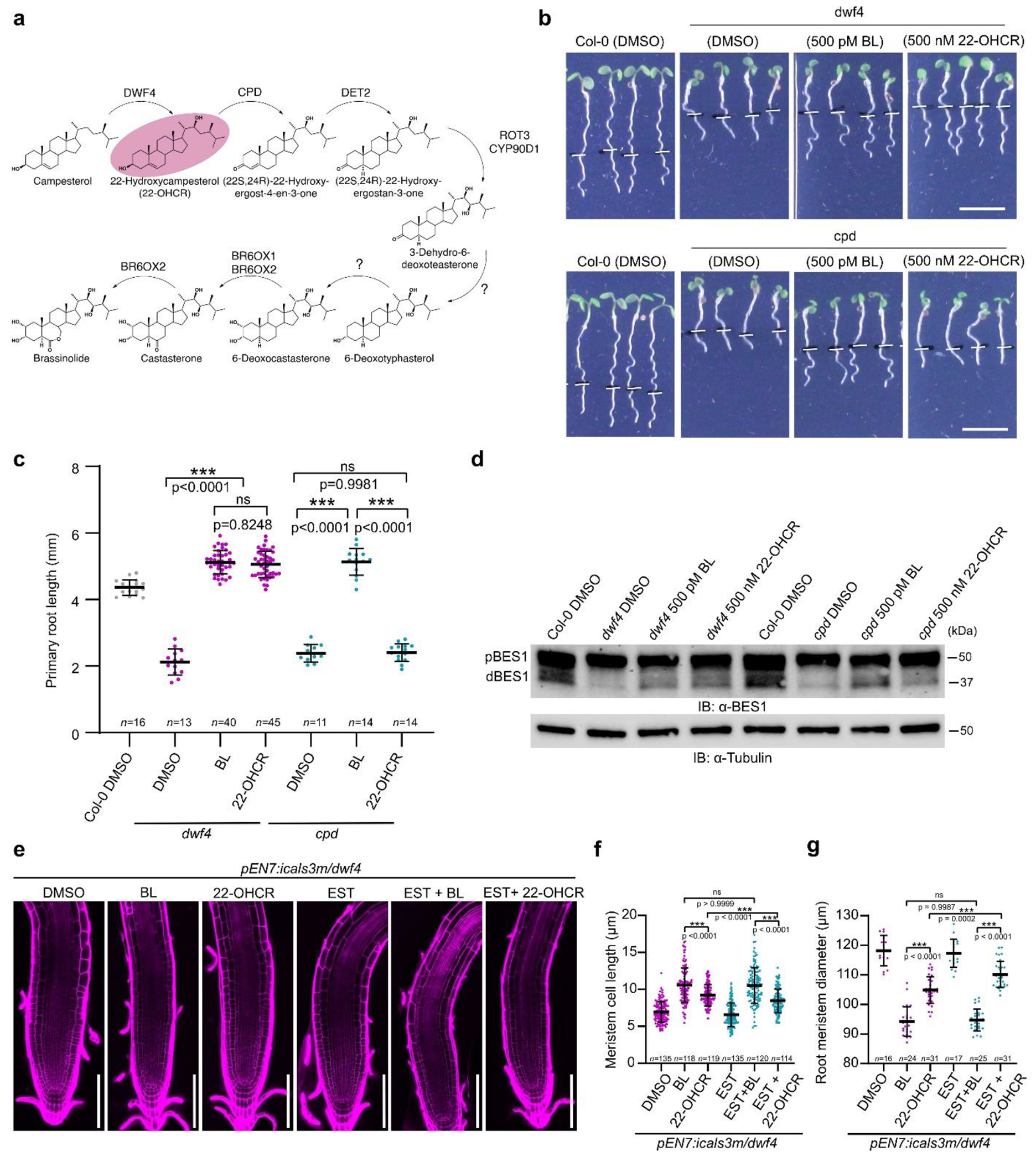
22-hydroxycampesterol (22-OHCR) is an inactive BR precursor. **a**, BR biosynthetic pathway with all known enzymes and their presumed position within the pathway. The position of 22-OHCR is highlighted. **b**, 22-OHCR rescued the root phenotype of *dwf4*, but not of *cpd*. Five-day-old *Arabidopsis* wild type (Col-0), *dwf4*, and *cpd* seedlings were transferred to agar medium containing BL (500 pM), 22-OHCR (500 nM), and DMSO (mock), and imaged after 24 h. Root tips were marked immediately after the transfer (white bars). Scale bars, 5 mm. **c**, Quantification of the primary root length of Col-0, *dwf4* and *cpd* in (**b**). Horizontal and error bars represent the means and the s.d., respectively. *n*, number of roots analyzed. The significant differences between the wild type (Col-0) and the mutants were determined with one-way analysis of variance (ANOVA) and Tukey’s multiple comparison tests. *** *P* < 0.001, ** *P* < 0.01 and * *P* < 0.05. **d**, Phosphorylation status of BES1 detected by immunoblotting (IB) with the α-BES1 antibody in seedlings in (**b**). Tubulin detected with the α-tubulin antibody was used as loading control. pBES1, phosphorylated BES1, dBES1, dephosphorylated BES1. **e**, Confocal images of 6 day-old root tips of *pBES1:gBES1-GFP/pEN7:icals3m/dwf4 Arabidopsis* seedlings stained with PI. Scale bars, 100 μm. **f,g**, Quantification of the meristem cell length (**f**) and root meristem diameter (**g**) of roots shown in (**e**). Horizontal and error bars represent the means and s.d., respectively. *n*, number cells. The significant differences were determined with one-way analysis of variance (ANOVA) and Tukey’s multiple comparison tests. *** *P* < 0.001, ** *P* < 0.01, and * *P* < 0.05. For **b,c,d,e,f,g**, the experiment was repeated twice with similar results.

**Fig. 2 |:**
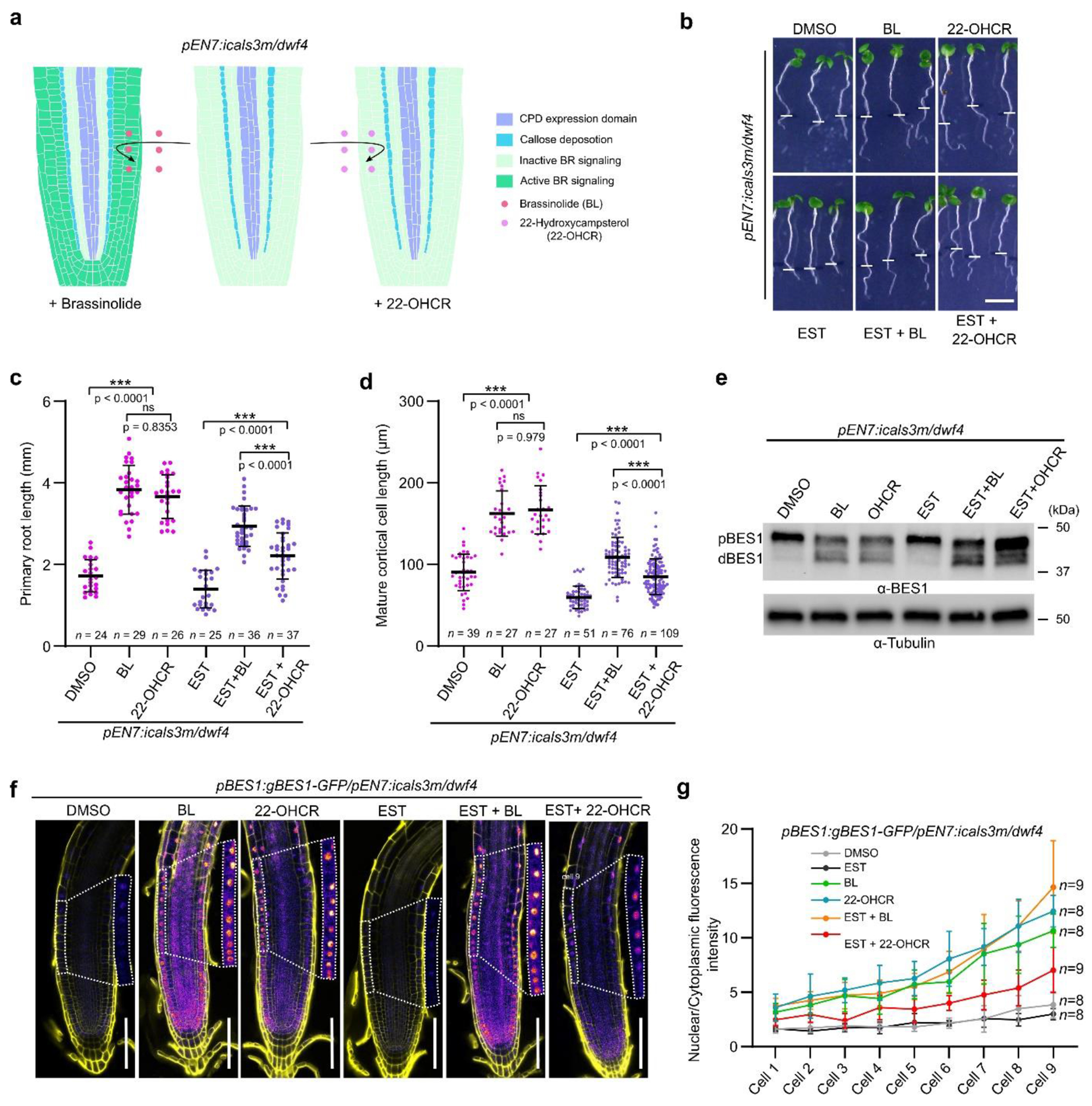
Brassinolide (BL), but not its precursor 22-hydroxycampesterol (22-OHCR) rescues *dwf4* mutants after PD closure. **a**, Schematic representation of different complementation principles by BL and 22-OHCR of the *dwf4* root upon callose deposition in the endodermis. **b**, The root growth phenotype of *pEN7:icals3m/dwf4* plants in response to exogenous BL and 22-OHCR upon callose induction. Four-day-old seedlings were transferred to agar medium containing either DMSO (mock) or estradiol EST (5 μM) for 24 h. Next, seedlings grown on DMSO were transferred to agar medium containing DMSO, BL (500 pM) or 22-OHCR (500 nM) (upper panel). Seedlings grown on EST were transferred to agar medium containing EST (5 μM), EST (5 μM) + BL (500 pM) and EST (5 μM) + 22-OHCR (500 nM) (lower panel) and grown for 24 h. Root tips were marked immediately after the transfer (white bars). Scale bar, 5 mm. **c,d**, Quantification of the primary root (**c**) and the mature cortical cell (**d**) lengths from (**b**). Horizontal and error bars represent the means and s.d., respectively. *n*, number of roots used in (**c**) and cells used in (**d**). The significant differences between the transgenic lines and the wild type (Col-0) control were determined with one-way analysis of variance (ANOVA) and Tukey’s multiple comparison tests. *** *P* < 0.001, ** *P* < 0.01, and * *P* < 0.05. **e**, Phosphorylation status of BES1 detected by immunoblotting (IB) with α-BES1 antibody in root tips. Tubulin detected with α-tubulin antibody was used as loading control. pBES1, phosphorylated BES1, dBES1, dephosphorylated BES1. **f**, Confocal images of 6 day-old root tips of *pBES1:gBES1-GFP/pEN7:icals3m/dwf4 Arabidopsis* seedlings treated as in (**b**). Cell walls were stained with propidium iodide (PI). Nine cells used for florescence intensity measurements in (**g**) are shown in insets (1.5x enlarged). In inset panels, only GFP signal is shown. Brightness and contrast were equally adjusted for each treatment. Scale bars, 100 μm. **g**, Quantification of nuclear/cytoplasmic BES1-GFP fluorescence intensity ratio of epidermal cells in the root transition zone (nine cells from insets panels in **f**). Error bars represent s.d. *n*, number of roots used for each treatment. **For b**,**c**,**d**, experiments were performed in three and for **e**,**f**,**g**, in two repeats with a similar results.

We hypothesized that the impaired BR signaling in different PD mutants was due to an altered mobility of BR precursors. To test this assumption, we employed BR precursor feeding experiments with 22-hydroxycampesterol (22-OHCR) (**1**) (Supplementary Note 1), which is the product and the substrate of DWF4 and CPD enzymes, respectively (Extended Data Fig. 4a). As expected, exogenous 22-OHCR (500 nM) was able to rescue the root growth of *dwf4* and to dephosphorylate BES1 in this mutant, similarly to BL (500 pM), whereas it was inactive when applied to the *cpd* mutant (Extended Data Fig. 4b–d). Next, the inducible *pEN7:icals3m* construct was introduced into the *dwf4* mutant to conditionally perturb PD permeability in the endodermis. We postulated that after induction of callose deposition at PD in the endodermis, exogenous BL would reach epidermal and cortical cell layers, where BR signaling is sufficient to rescue the root growth of BR-related mutants^31^ (Fig. 2a). Conversely, the inactive BR precursor 22-OHCR would need to move to the stele, where the CPD enzyme is expressed, to be converted into the following BR precursor and, finally, to BL to rescue *dwf4* root growth. In a scenario in which BRs move via the PD, callose-induced PD blockage in the endodermis would interrupt the biosynthetic pathway and exogenous 22-OHCR would not rescue the *dwf4* defects (Fig. 2a). Consistent with this hypothesis, we found that, without callose induction, both exogenous 22-OHCR (500 nM) and BL (500 pM) rescued the root growth defects of *pEN7:icals3m/dwf4* plants, including primary root and mature cortical cell lengths (Fig. 2b–d). However, after blocking PD in the endodermis, 22-OHCR was less effective in rescuing the *dwf4* root growth defects than BL (Fig. 2b–d and Extended Data Fig. 4e–g ).

To further corroborate the different capacities of exogenous BL and 22-OHCR to rescue *dwf4* when PD were closed in the endodermis, we evaluated BR signaling by immunoblot analysis of the phosphorylation status of BES1. Because the *EN7* promoter is only active in the root tip, we collected root tips for immunoblotting. In accordance with the observed root phenotypes, BR signaling was less efficiently recovered by 22-OHCR than by BL after callose induction (Fig. 2e). Then, we introduced the *pBES1:gBES1-GFP* construct in *pEN7:icals3m/dwf4* plants and quantified the nuclear accumulation of BES1-GFP in the root epidermis as a BR signaling read-out. As predicted, BR signaling in the mock-treated *pBES1:gBES1-GFP/pEN7:icals3m/dwf4* seedlings was enhanced by exogenous BL and 22-OHCR with equal efficiency (Fig. 2f,g), but it was only partially complemented by 22-OHCR in the estradiol-treated seedlings (Fig. 2f, g).

Taken together, these results suggest that PD closure renders BR biosynthesis inefficient, possibly due to the necessity for BR precursors to be exchanged through PD between adjacent root cell files.

### Direct visualization of BR movement.

**Extended Data Fig. 5|:**
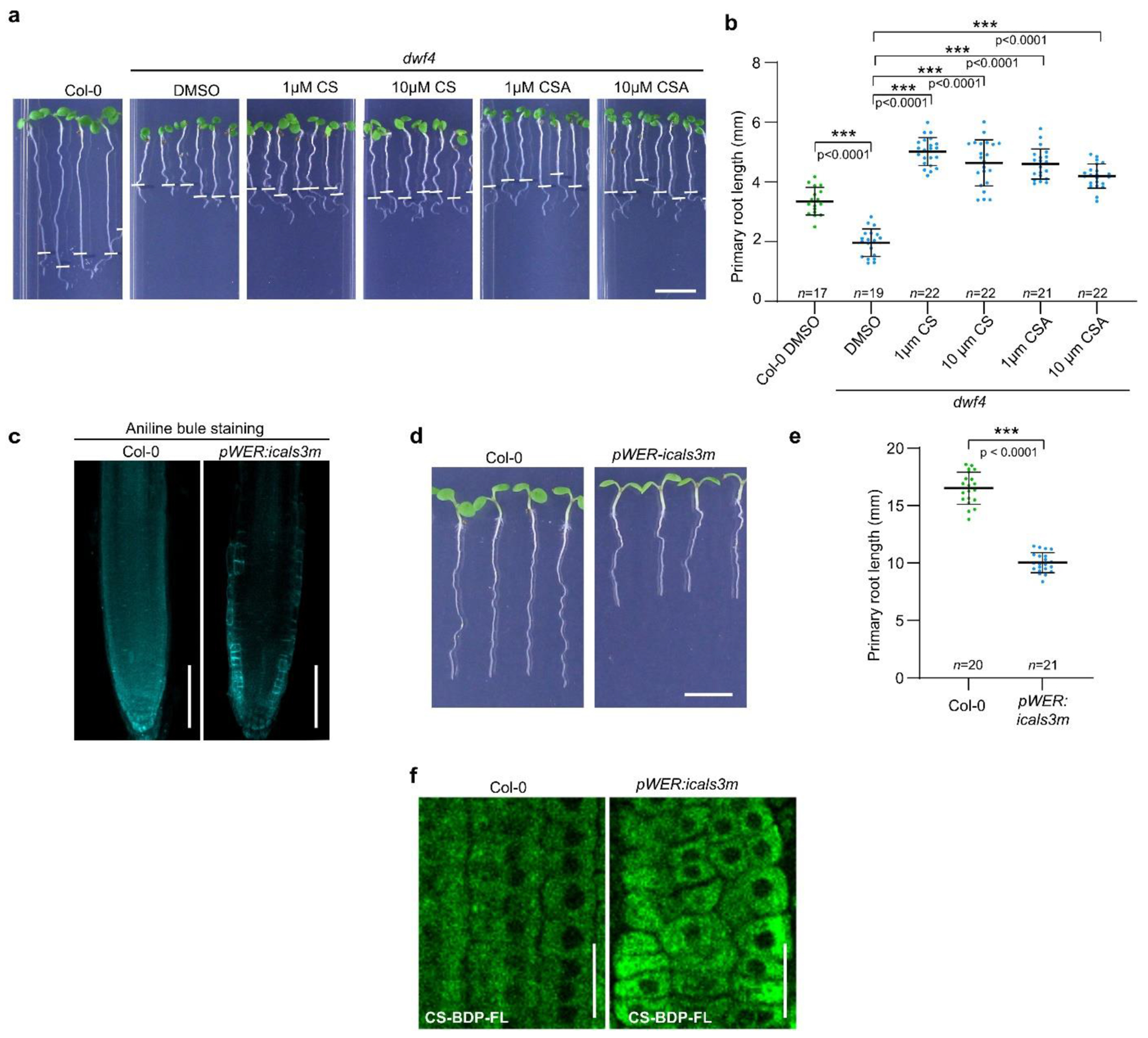
Biological activity and uptake of castasterone-alkyne (CSA). **a**, CSA retains the biological properties of castasterone (CS). Five-day-old seedlings were transferred to agar media containing different concentrations of CS or CSA as indicated and DMSO (mock) for 24 h. Root tips were marked immediately after the transfer (white bars). Scale bar, 5 mm. **b**, Quantification of primary root length in (**a**). Horizontal and error bars represent the means and the s.d., respectively. *n*, number of roots analyzed. The significant differences were determined with one-way analysis of variance (ANOVA) and Tukey’s multiple comparison tests. *** *P* < 0.001, ** *P* < 0.01, and * *P* < 0.05. **c**, Aniline blue staining of callose deposition in 6-day-old *pWER:icals3m* plants. Wild type (Col-0) was used as control (left panel). Scale bars, 100 μm. **d**, Phenotypes of 6-day-old wild type (Col-0) and *pWER:icals3m* seedlings. Scale bar, 5 mm. **e**, Quantification of primary root length in (**c**). Horizontal and error bars represent the means and the s.d., respectively. *n*, number of roots analyzed. The significant differences were determined with two-tailed Student’s unpaired *t*-test analysis. *** *P* < 0.001, ** *P* < 0.01, and * *P* < 0.05. **f**, Accumulation of CS-BDP-FL signal in in the epidermal cells of Col-0 and *pWER:icals3m* six-day-old seedlings. Scale bars, 25 μm. For **a,b,d,e**, the experiment was repeated twice with similar results.

**Fig. 3 |:**
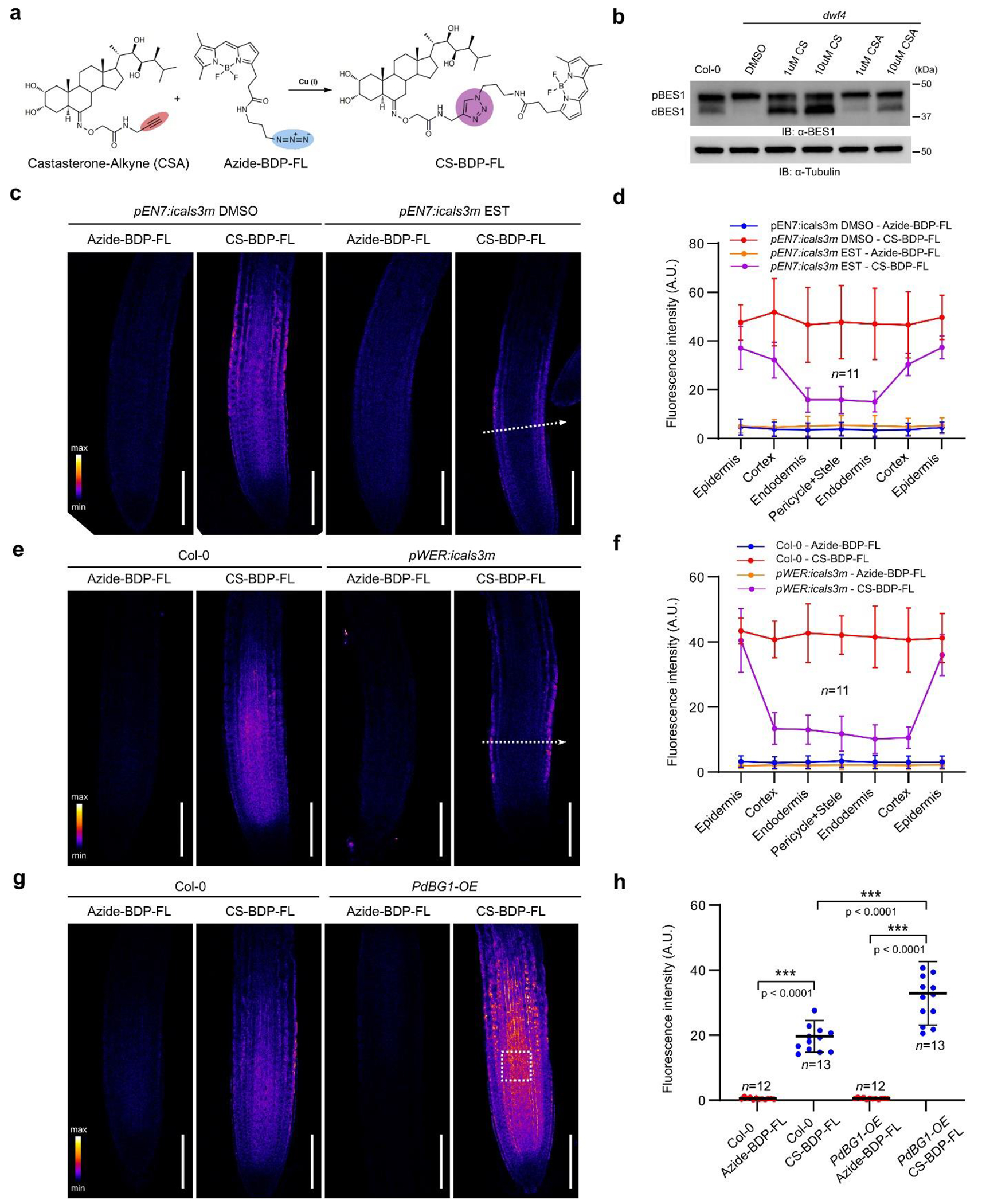
Localization of castasterone-alkyne (CSA) in *Arabidopsis* root tips. **a**, Chemical compounds used in the bioorthogonal reaction. **b**, CSA is retaining the biological properties of castasterone (CS). Phosphorylation status of BES1 detected with immunoblot (IB) and α-BES1 antibody. Tubulin detected with the α-tubulin antibody was used as loading control. pBES1, phosphorylated BES1, dBES1, dephosphorylated BES1. Five-day-old wild type *Arabidopsis* and *dwf4* mutant seedlings were transferred to agar medium containing CS or CSA at the indicated concentrations and DMSO (mock) for 24 h. **c**, Blocking PD in the endodermis arrests CS-BDP-FL signal in epidermal and cortical cells. Five-day-old seedlings were transferred to agar medium containing either EST (5 μM) or DMSO (mock) for 24 h. Next, seedlings from each treatment were divided into two and transferred to liquid media containing either CSA (20 μM) or DMSO (mock) for 4 h, followed by bioorthogonal reaction. **d**, Fluorescence intensity quantification along the white dashed line in (**c**), positioned at 200 μm away from the root tip shown in (**c**). **e**, Blocking the PD in epidermal cells arrests CS-BDP-FL signal accumulation in the epidermis. Six-day-old seedlings were incubated in liquid medium with either CSA (20 μM) or DMSO (mock) for 4 h followed by a bioorthogonal reaction. **f**, Quantification of fluorescence intensity in (**e**), positioned at 200 μm away from the root tip shown in (**e**). **g**, Highly permeable PD increase the CSA uptake. Six-day-old PdBG1-OE plants were treated as in (**e**). **h**, Quantification of fluorescence intensity in (**g**), in which 50 × 50 μm^2^ area, 200 μm away from root tip (white dashed box in **g**) was used. The significant differences were determined with one-way analysis of variance (ANOVA) and Tukey’s multiple comparison tests. *** *P* < 0.001, ** *P* < 0.01, and * *P* < 0.05. Scale bars, 100 μm ((**c,e,g**). Horizontal bars in (**h**) represent the means and error bars in ((**d,f,h**) represent the s.d. *n*, number of roots (**d,f,h**). A. U., arbitrary units. For **b,e,f** the experiment was repeated two times, for **c,d,** four times and for **g,h**, three times with similar results.

To validate the PD-mediated intercellular transport of BRs and directly visualize BRs in *Arabidopsis* roots, we used a bioorthogonal chemistry approach. To this end, castasterone-alkyne (CSA) (**2**) was synthesized by introduction of an alkyne group attached to a linker connected to the C-6 position of the castasterone (CS) molecule (Fig. 3a and Supplementary Note 2), which is the direct precursor of BL (Extended Data Fig. 4a). CSA can be visualized after cycloaddition or a click reaction between its alkyne and the azide group of the commercially available green-emitting fluorescent dye azide-BODIPY-fluorescein (azide-BDP-FL)^32^ to generate CS-BDP-FL (**3**) (Fig. 3a). Although slightly less potent than CS, CSA retained biological activity, because it rescued the short root phenotype of the *dwf4* mutant and induced BES1 dephosphorylation (Fig. 3b and Extended Data Fig. 5a,b). Subsequently, a click reaction was carried out on transgenic *Arabidopsis* lines with perturbed PD permeability. Initially, the *pEN7:icals3m* line was utilized, which has increased callose deposition (Fig. 1a) and closed PD in the endodermis after estradiol induction. In the absence of CSA, no signal was detected in the mock- and estradiol-treated seedlings (Fig. 3c), indicating that the azide-BDP-FL fluorescent dye did not bind non-specifically, whereas in the presence of CSA and after a click reaction with azide-BDP-FL the CS-BDP-FL signal occurred only in epidermal and cortical cells in estradiol-treated roots, but it was ubiquitous in all cell files in non-induced roots (Fig. 3c,d). These findings suggest that closed PD in the endodermis block the CSA movement from the cortex to the inner tissues.

Consequently, a similar click reaction was carried out with a line expressing *icals3m* from the *WEREWOLF (WER)* promoter^33^, *pWER:icals3m*. This line specifically accumulated callose in epidermal cells, presumably blocking PD in this tissue (Extended Data Fig. 5c), and caused root growth inhibition (Extended Data Fig. 5d,e). In the root tips of the *pWER:icals3m* seedlings treated with CSA followed by a click reaction, the fluorescent CS-BDP-FL signal accumulated preferentially in epidermal cells when compared to the Col-0 control (Fig. 3e,f), implying that PD closure in the epidermal cells is sufficient to block CSA from moving to the inner tissues. Notably, the uptake of CSA in the epidermal cells was not compromised by extensive callose deposition (Extended Data Fig. 5f), hinting that the lack of fluorescent signal in inner tissues of *pWER:icals3m* line, is due to the compromised symplastic transport and not impaired compound uptake. Finally, CSA click chemistry experiments were done with the PdBG1-OE plants^30^, which have open PD. The accumulation of the fluorescent signal in all cell layers was higher than that of the Col-0 control (Fig. 3g,h), suggesting an enhanced accumulation of CSA in the root when the PD aperture is increased. In conclusion, bioorthogonal chemistry allowed the direct visualization of a bioactive BR precursor in plant tissues, confirming the involvement of PD in intercellular BR transport.

### BRs modulate PD permeability.

**Extended Data Fig. 6|:**
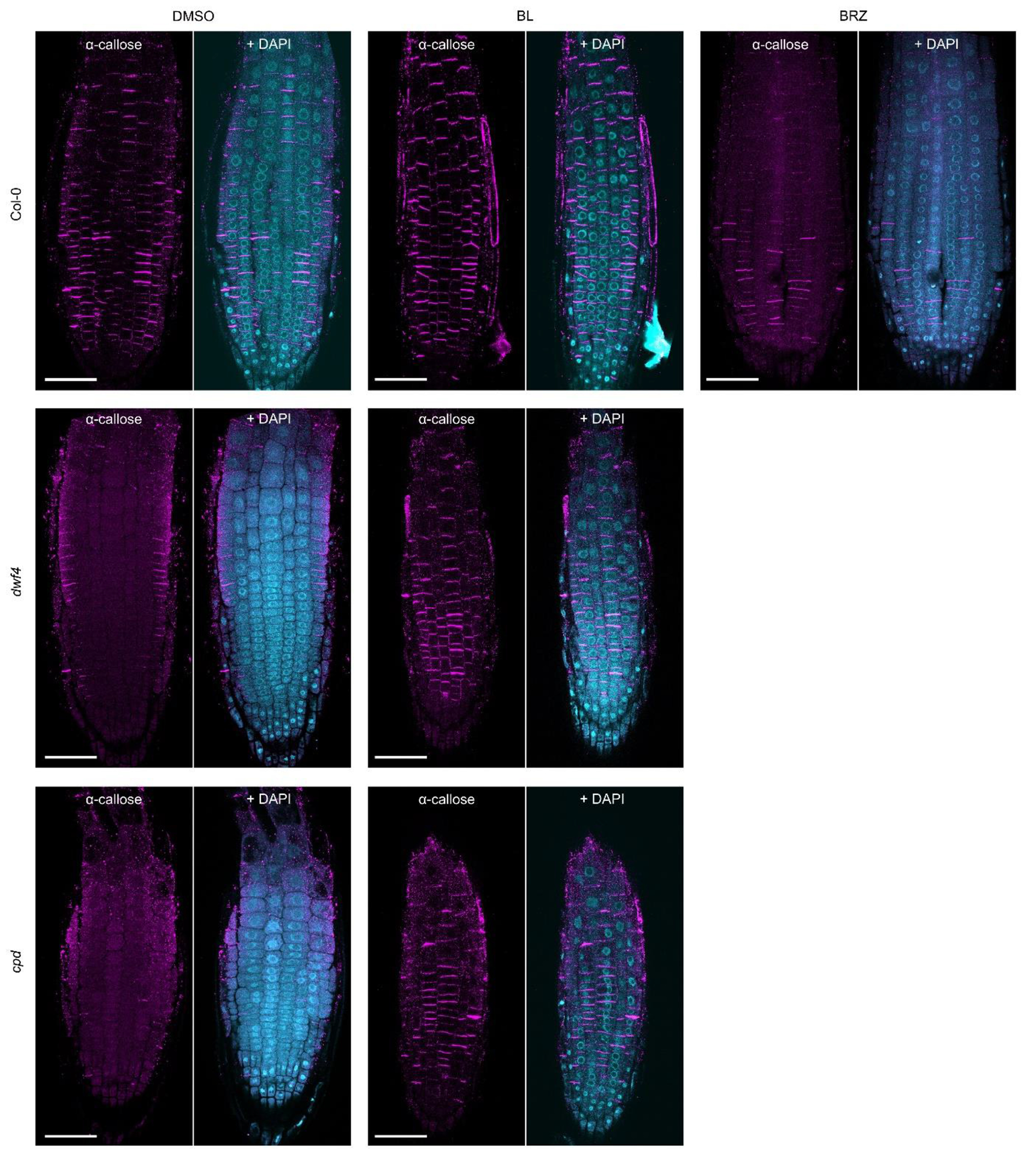
BRs positively regulate callose deposition in roots. Callose immunostaining of *Arabidopsis* wide type (Col-0), *dwf4*, and *cpd* roots. Four-day-old seedlings were transferred to agar medium containing brassinolide (BL) (200 nM), brassinazole (BRZ) (1 μM) and DMSO (mock) for 24 h. Cell nuclei were stained by 4′,6-diamidino-2-phenylindole (DAPI). Epidermal cell layers are shown. Scale bars, 50 μm. The same experimental material was used to quantify callose deposition at PD in Figure 4b.

**Fig. 4 |:**
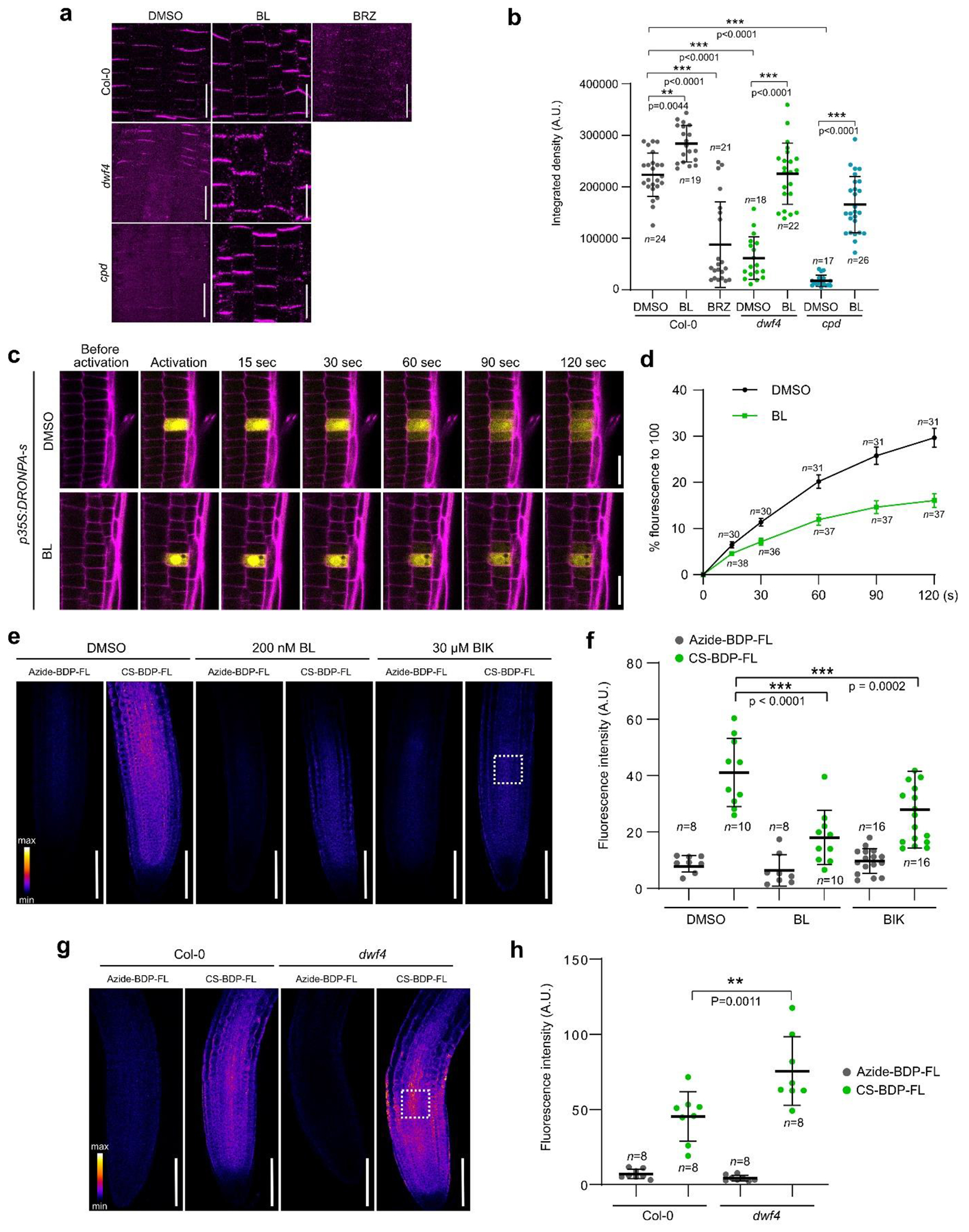
BRs regulate PD permeability. **a**, Callose immunostaining of *Arabidopsis* wide type (Col-0), *dwf4*, and *cpd* roots using α-callose antibody. Four-day-old seedlings were transferred to agar medium containing brassinolide (BL) (200 nM), brassinazole (BRZ) (1 μM) and DMSO (mock) for 24 h. Scale bars, 20 μm. **b**, Quantification of callose intensity at the PD in (**a**). Callose signal at the cell plate was excluded from measurements. **c**, DRONPA-s movement after activation in a single meristematic cell of the root. Confocal images of six-day-old root meristems stained with propidium iodide (PI) are shown. Roots were treated either with mock or BL (200 nM) for 24 h prior to imaging. Scale bars, 20 μm. **d**, Normalized values of the mean fluorescence intensity of the DRONPA-s are extracted from the adjacent cells next to the activated cell. For each time point n > 15. Error bars represent standard error of the mean (SEM). **e**, Reduction of the castasterone-alkyne (CSA) uptake after BL and bikinin (BIK) treatment. Five-day-old seedlings were transferred to agar medium containing BL (200 nM), BIK (30 μM) or DMSO (mock) for 24 h, followed by the bioorthogonal reaction to form CS-BDP-FL. **f**, Quantification of fluorescence intensity of images in (**e**) in the boxed region of interest positioned 200 μm away from root tip. **g**, *dwf4* seedlings exhibited an increased CSA uptake capacity. The bioorthogonal reaction was done in six-day-old *Arabidopsis* seedlings of Col-0 and *dwf4*. **h**, Quantification of fluorescence intensity of images in (**g**) in the boxed region of interest positioned 200 μm away from root tip. Horizontal and error bars (**b,f,h**) represent the means and s.d., respectively. *n*, number of roots (**b,f,h**) The significant differences were determined with one-way analysis of variance (ANOVA) and Tukey’s multiple comparison tests. *** *P* < 0.001, ** *P* < 0.01, and * *P* < 0.05 for (**b**,**f,h**). Scale bars, 100 μm (**e,g**). A. U., arbitrary units. For **a,b**, experiments were performed in four repeats, for **c,d,e,f**, in three repeats and for **g,h**, in two repeats with similar results.

As BRs are transported through the PD, we asked if BRs could, in turn, modulate PD permeability. To investigate this hypothesis, we visualized callose deposition by means of a callose-specific antibody in wild type Col-0 plants treated with either BL (200 nM) or the BR biosynthesis inhibitor brassinazole (BRZ) (1 μM) for 24 h. The immunostaining revealed that BL-treated seedlings exhibited increased callose deposition, whereas the callose levels were significantly reduced after BRZ treatment (Fig. 4a,b and Extended Data Fig. 6). Consistently, the BR deficient *dwf4* and *cpd* mutants had almost no callose deposition in their root meristems, but the observed callose deficiency was rescued by exogenous BL (Fig. 4a,b and extended Data Fig. 6). These results indicate that BRs can modulate PD permeability and that high and low BR signaling would induce closure and opening of PD, respectively.

**Extended Data Fig. 7|:**
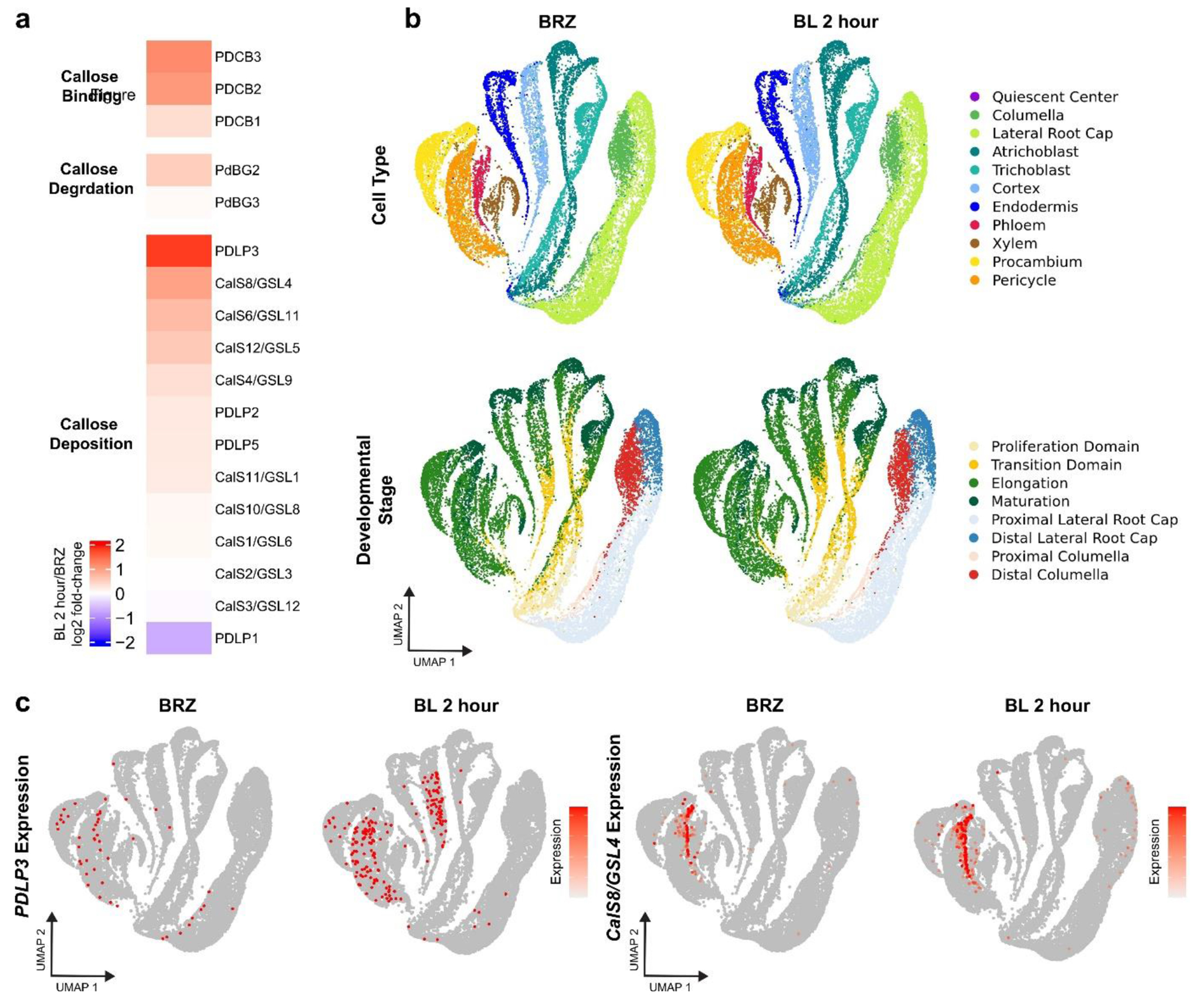
Expression levels of callose deposition-related genes are positively regulated by BRs. **a**, Heatmap showing relative expression of PD-related genes across all cells from publicly available brassinolide (BL) 2 hour scRNA-seq compared to brassinazole (BRZ) scRNA-seq. Color represent log2 fold-change of BL 2 hour versus BRZ. **b**, Two-dimensional uniform manifold approximation and projection (UMAP) embedding of BRZ and BL 2 hour treated cells from scRNA-seq dataset^36^. Colors indicate cell type (top) or developmental stage (bottom). **c**, *PDLP3* and *CalS8/CSL4* expression in BRZ and BL 2 hour scRNA-seq. The color scale on the UMAP projection represents log normalized, corrected UMI counts

To explore whether the symplastic BR transport is regulated by BR signaling, we performed photoactivation of cytosolic DRONPA-s, a reversibly switchable photoactivatable fluorescent protein in single root cells and monitored the spread of the fluorescent signal to the surrounding cells^34^ (Fig. 4c). We observed significantly faster spread of the signal in mock-treated Col-0 plants in comparison to BL-treated ones (Fig. 4d), demonstrating the ability of BRs to decrease PD permeability. To directly observe the distribution of CSA under conditions of perturbed BR signaling, click chemistry was utilized. As anticipated, exogenous BL significantly reduced the accumulation of CSA in Col-0 roots when compared with the mock control (Fig. 4e,f). Conversely, CSA accumulation in the root meristems of *dwf4* seedlings was stronger than in Col-0 roots (Fig. 4g,h). To examine whether the reduced CSA accumulation is a consequence of increased BR signaling levels and not competition with BL, we tested CSA accumulation after bikinin (BIK) treatment, which can activate BR signaling downstream of the BRI1 receptor^35^. Similar to BL, BIK application reduced CSA accumulation in the treated plants (Fig. 4e,f). The BR-induced callose turnover and control of PD permeability is likely under transcriptional control, since BR signaling positively regulates expression levels of several callose deposition-related genes in the Arabidopsis root^36^ (Extended Data Fig.7 and Supplementary Table 1).

In summary, our findings demonstrate the existence of an additional BR signaling regulatory feedback loop that acts at the PD permeability level and ensures optimal BR biosynthesis by modulating BR movement.

## Discussion

Polyhydroxylated steroidal molecules are utilized as signaling molecules in both mammals and plants to control a plethora of developmental processes^37–39^. Although these molecules share similar structures, they impact gene expression differently. For example, in mammals, steroidal hormones act in an endocrine manner, namely they are synthesized in glands, transported by specialized protein carriers, and after release into the target cell cytoplasm, they bind to intracellular receptors^11^. In plants, a family of plasma membrane-localized receptors, including BRI1 and its homologs BRI1-LIKE1 (BRL1) and BRL3, perceive locally produced BRs in the apoplast^40^, because plant steroidal hormones do not undergo long-distance transport^12^. The textbook knowledge suggests that steroid hormones can freely diffuse across biological membranes, due to their hydrophobicity. However, the simple diffusion theory was challenged by finding that the release of the steroid hormone ecdysone in *Drosophila melanogaster* requires ATP-binding cassette (ABC) transporter-mediated vesicle loading and calcium-mediated vesicle exocytosis^41^, and the cellular uptake of ecdysone needs a membrane solute carrier (SLC) transporter. In addition, molecular dynamics simulation experiments demonstrated that the flip-flop transitions of steroids, and hence, their ability to diffuse across lipid membranes, are determined by the number of hydroxyl groups in the molecule^42^. Here, we confirmed that the BR biosynthetic enzymes are localized in the ER membrane, which is the most likely place of the BR biosynthesis. As BR biosynthetic enzymes are expressed in a non-overlapping fashion along the radial axis of *Arabidopsis* roots, exchange of precursors between cells of different cell files is necessary for completion of bioactive BR synthesis. Given that BRs are hydrophobic molecules, it is unlikely that they freely move *via* an apoplastic route between neighboring cells. Alternatively, PD-mediated symplastic transport can ensure movement of steroid molecules in plants. This pathway enables cell-to-cell exchange of nutrients, hormones, RNAs, proteins, metabolites, and viruses^15–19^. Therefore, we speculated that the intermediates or bioactive BRs are transported through the PD after synthesis in the PD vicinity. In the scenario of PD-mediated BR transport, perturbation of PD permeability should affect BR signaling. Examination of *Arabidopsis* plants with altered PD permeability revealed that BR signaling was modulated to certain extent in plants with perturbed PD permeability, possibly due to the reduced or enhanced ability to transport BRs through the PD. Nevertheless, BR signaling levels were not drastically changed when PD conductivity was altered. The reason for this might be the pleiotropic nature of PD mutants, and the fact that the molecules which negatively regulate BR signaling^43^, are also transported via PD. Additionally, changes in the cell wall composition of PD mutants might trigger BR signaling without the ligand binding^44^ and mask reduced hormone synthesis. Whether the BR biosynthetic machinery is associated with PD, and what physiological relevance this association might have, remains to be tested in details in future studies.

To further demonstrate the PD-mediated BR movement, we used precursor feeding experiments. Conditionally disrupting cell-to-cell communication specifically in the endodermis prevented the rescue of *dwf4* mutants with their exogenous product, 22-OHCR. Hence, we concluded that closed PD in mid-positioned root cell files block this BR precursor movement and prevent it from reaching inner tissues where it can be converted to downstream BR precursors. Nevertheless, it seems that transport of 22-OHCR was not completely blocked by the PD constriction, leaving the possibility for partial apoplastic movement of the hormone. Currently, no tools are available that allow monitoring the BR distribution or movement in plant tissues, in contrast to other plant hormones^45^. Alexa Fluor 647-castasterone (AFCS) is the only existing bioactive fluorescently labeled BR that could be used to track BR movements in living cells^46^, but this chemical probe might be problematic because of the bulky fluorophore. Therefore, we took a bioorthogonal chemistry approach and developed the BR probe CSA that can be visualized with confocal microscopy after conjugation with azide-BDP-FL probe. Click chemistry allowed us to follow the CSA distribution in *Arabidopsis* roots under perturbed PD permeability conditions. When the endodermal PD were closed, CSA accumulated in epidermal and cortical cells and could not reach the stele, but when PD were blocked in the epidermis, the CSA was detected only in epidermal cells. This interesting finding implies that only epidermal cells of the root can directly uptake BRs and possibly other steroid-like compounds frequently used in plant research, such as estradiol. In addition, plants with an enhanced PD permeability accumulated more CSA in all cell files of the root. Together, these results demonstrate that the PD play a crucial role in the short-distance BR transport.

Maintenance and regulation of BR levels in cells are essential for plant growth and development. The feedback transcriptional regulation of key BR biosynthetic genes by direct BES1 and BZR1 binding to their promoters is one of the well-known mechanisms for retaining BR homeostasis^5^. Intriguingly, in *Arabidopsis* roots, the expression domains of BR biosynthetic enzymes are separated. We hypothesize that this spatial organization of the biosynthetic pathway allows a more flexible control of hormone biosynthesis through precursor transport regulation. Indeed, the callose immunostaining together with click chemistry-based visualization of BRs revealed that intracellular BR content, probably through BR signaling, regulates PD permeability. Presumably, control of the callose regulating turnover, and, hence, BR mobility, can optimize BR biosynthesis and signaling, thus creating a negative feedback regulation loop between PD permeability and intracellular BR content. Nevertheless, how BR signaling impacts callose production and degradation at PD, remains to be investigated. Recently, the callose synthase gene, *GLUCAN SYNTHASELIKE8 (GSL8)* with a function in plant defense has been reported as a direct target of BES1, and its expression to be induced by BRs^47^. Our analysis of existing BR response single-cell RNA-sequencing datasets^36^ revealed that two other genes implicated in callose turnover regulation in the Arabidopsis root , PDLP3^48^ and GSL4^49^, are upregulated after BR treatment.

**Fig. 5 |:**
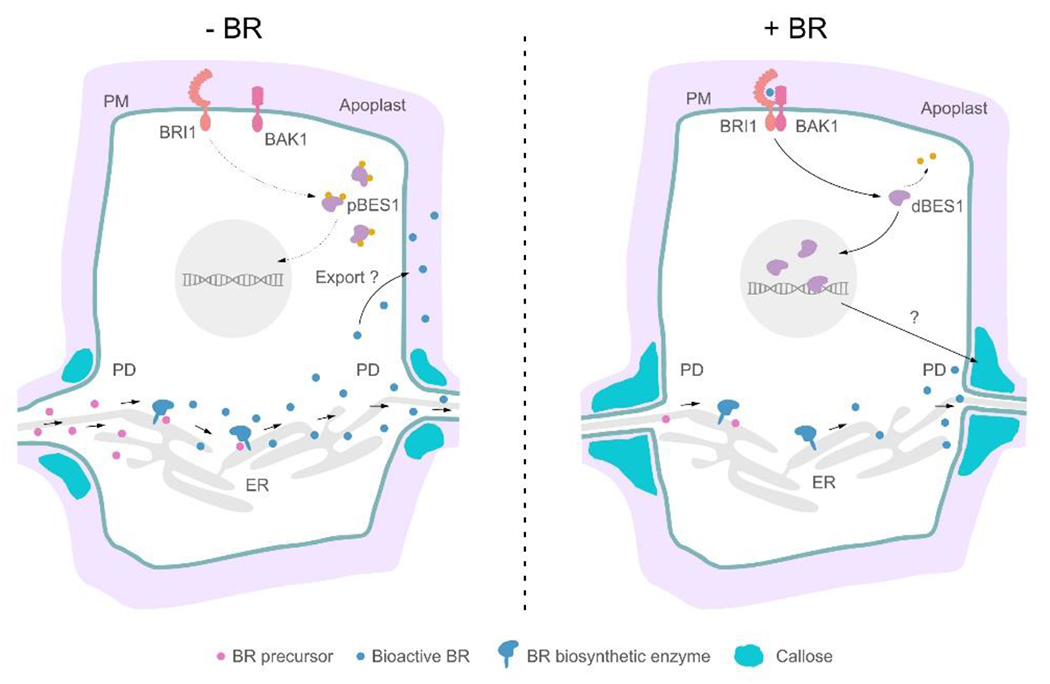
PD-mediated transport and homeostasis of BR biosynthesis and signaling. BR biosynthetic enzymes are expressed in neighboring cells, requiring the exchange of BR intermediates through PD to produce bioactive BRs. Once synthetized, BRs exit the cell via an unknown mechanism and reach the apoplast (left panel). Once in the extracellular matrix, bioactive BRs bind to the BRI1 receptor and coreceptor BAK1 and initiate a signaling cascade that leads to dephosphorylation of the BES1 transcription factor. BES1 can then enter the nucleus and initiate transcriptional responses. Elevated levels of BRs lead to increased callose deposition and decreased PD permeability, possibly via BR signaling-initiated transcriptional regulation. Restricted BR precursor movements decrease hormone production and contribute to optimal BR signaling level maintenance (right panel). pBES1, phosphorylated BES1; dBES1, dephosphorylated BES1; ER, endoplasmic reticulum; PM, plasma membrane.

Collectively, our results show that BR precursors are transported through PD after their biosynthesis in the PD-neighboring ER membrane to complete BR biosynthesis and to produce bioactive BRs (Fig. 5). In turn, elevated BR levels can alter callose deposition and decrease PD permeability, possibly through transcriptional regulation initiated by BR signaling, thereby reducing hormone production by restricting the movement of BR precursors. In contrast, low intracellular BR levels lead to decreased callose deposition and increased PD permeability, with enhanced hormone production as a consequence. This negative feedback loop ensures the precise amount of bioactive BRs that is beneficial to plant growth and development. Our work reveals a thus far unknown BR transport pathway in plants, and expands the regulatory mechanisms of hormone homeostasis.

## Methods

### Plant materials and growth conditions.

The *Arabidopsis thaliana* (L.) Heynh., accession Columbia-0 (Col-0), was used in all experiments. Seeds were surface-sterilized with sterilization buffer (80% [v/v] ethanol, 20% [v/v] sodium hypochlorite), stratified for 2 days in the dark at 4°C and grown vertically on half-strength Murashige and Skoog (½MS) 1% agar (w/v) plates supplemented with 1% (w/v) sucrose at 22°C, with a 16 h/8 h light/dark photoperiod. The following mutant lines were used *dwf4-102*^50^, *cpd*^51^, *pDWF4:DWF4-GFP/dwf4*^6^, *pROT3:ROT3-GFP/rot3*^6^, *pCPD:CPD-GFP/cpd*^6^, *pBR6OX2:BR6OX2-GFP/br6ox1;br6ox2*^6^, *pBES1-gBES1-GFP*/Col-0^6^, *pEN7:icals3m*/Col-0^27^ and *p35S:PdBG1-mCitrine*/Col-0^30^. Primers used for genotyping are listed in Supplementary Table 2. The lines *pEN7:icals3m/dwf4* and *pBES1:gBES1-GFP/pEN7:icals3m/dwf4* were obtained by crossing *pEN7-icals3m* with *dwf4-102* and *pEN7:icals3m/dwf4* with *pBES1:gBES1-GFP*/Col-0, respectively.

### Plasmid construction and generation of transgenic lines.

To generate the *p35S:C4H-mCherry* and *pUBQ10:PDLP1-mCherry* constructs, the genomic fragments of *C4H* (AT2G30490) and *PDLP1* (AT5G43980) were amplified by PCR and cloned into pDONR221 (Thermo Fisher Scientific). The resulting entry clones were recombined with the cauliflower mosaic virus [*CaMV*]*35S* promoter in pDNORP4-P1R and with the *UBIQUITIN10* promoters in pDNORP2R-P3-mCherry for *C4H* and *PDLP1*, respectively into the pH7m34GW^52^ destination vector by an LR reaction. The resulting constructs were used for transient expression in tobacco and generation of transgenic plants by introducing them into *pDWF4:DWF4-GFP/dwf4* and *pROT3:ROT3-GFP/rot3* plants by *Agrobacterium tumefaciens*-mediated transformation according to the floral dip protocol^53^. To generate *pWER:icals3m*, pDONRP4-P1R-WER (obtained from NASC set 2106366; https://arabidopsis.info/StockInfo?NASC_id=2106366) was recombined with *icals3m* in pDONRP1-P2 and without in pDONRP2R-P3 into the pB7m34GW^52^ destination vector by a LR reaction. The construct was transformed into Col-0. To generate *pCPD:PDLP5-BFP/pCPD:CPD-GFP/cpd* and *pCPD:PDLP5-BFP/pSCR:CPD-mCherry/cpd* lines, the genomic fragment of *PDLP5* was cloned into pDONR221 and combined in an LR reaction with pDONRP4-P1R-CPD^6^ and pDNORP2rP3-BFP in the destination vector PK8m34GW-FAST. The resulting constructs were introduced into *pCPD:CPD-GFP/cpd*^6^ and *pSCR:CPD-mCherry/cpd* translational lines. To generate *p35S:DWF4-GFP* and *p35S:ROT3-GFP* construct, the entry clones pDONR221-DWF4 and pDONR221-ROT3^6^ were put into pK7FWG2 destination vector by an LR reaction. To generate *p35S:MCTP3-GFP* construct, *MCTP3* (AT3G57880) was amplified by PCR and cloned into pDONR221 (Thermo Fisher Scientific). The resulting entry clone was put into pK7FWG2 destination vector by an LR reaction. The cloning primers are listed in Supplementary Table 2.

### Chemical treatments.

Brassinolide (BL) (OlChemIm Ltd.), brassinazole (BRZ) (TCI), β-estradiol (EST) (Sigma-Aldrich), Bikinin (BIK) (Sigma), 22-OHCR and CSA were kept at different stock concentrations in DMSO and were diluted 1000× to reach the final concentrations in the media. For the mock treatment, DMSO was at a final concentration of 0.1% (v/v). Specific treatments are described in the main text and/or figure legends.

### Immunoblot analysis.

For the BES1 immunoblot analysis of *pEN7:icals3m* seedlings, the experiments were done in triplicate. Five-day-old Col-0 and *pEN7:icals3m* seedlings were transferred to fresh ½MS medium agar plates containing DMSO or 5 μM EST. The root tips were collected for immunoblotting after 12 h of treatment. For the BES1 immunoblot analysis of *pCPD:PDLP5-BFP/pCPD:CPD-GFP/cpd* and *pCPD:PDLP5-BFP/pSCR:CPD-mCherry/cpd* lines, the experiments were done in triplicate. Six-day-old seedlings (root part) were collected for the BES1 assay. For the *p35S:PdBG1-mCitrine* line, experiments were done in duplicate. Six-day-old seedlings of *p35S:PdBG1-mCitrine* and Col-0 were collected for the BES1 assay. For the 22-OHCR activity tests, experiments were done in duplicate. Five-day-old Col-0, *dwf4*, and *cpd* seedlings were transferred to ½MS agar plates containing DMSO, BL (500 pM), or 22-OHCR (500 nM) for 24 h and collected for the BES1 assay. For the CSA activity test, experiments were done in duplicate. Five-day-old *dwf4* seedlings were transferred to fresh ½MS medium agar plates containing DMSO, CS (1 μM and 10 μM), or CSA (1 μM and 10 μM) and harvested after 24 h for the BES1 assay. All plant material was frozen in liquid nitrogen, ground by Retsch MM400, and homogenized in 100 μl of ice-cold homogenization buffer (1% [v/v] SDS, 25 mM Tris/HCl, pH 7.5, 150 mM NaCl, 10 mM DTT, and Roche Complete protease inhibitor [one tablet/10 ml]) and placed on ice for 30 min. The homogenates were centrifuged twice (10 min, 20, 000 ×*g*) at 4°C. After the addition of 4× lithium dodecyl sulfate and sample-reducing agent (10×), the samples were heated for 10 min at 70°C, centrifuged again, separated on 4-15% (v/v) SDS–polyacrylamide gel electrophoresis stain-free protein gel (Bio-Rad Laboratories), and blotted on Trans-Blot Turbo Mini PVDF Transfer Packs. Membranes were blocked at 4°C in 5% (v/v) skimmed milk (Difco). For immunodetection, the anti-BES1 antibody at 1:5000 was used as primary antibody and donkey anti-rabbit (Merck) at 1:10000 as the secondary antibody. For tubulin detection, the anti-tubulin (Abcam) at 1:5000 was used as primary antibody and sheep anti-mouse (Merck) at 1:10000 as secondary antibody. Proteins were detected by the ChemiDoc MP Imaging System (Bio-Rad Laboratories). For the BES1 dephosphorylation assay, the ratio of dephosphorylated BES1 to total BES1 proteins was quantified according to the signal intensity. Loading was adjusted to an equal level based on the amount of tubulin. Signal intensities were determined with Image Lab (Bio-Rad Laboratories).

### Synthesis of chemical compounds and bioorthogonal chemistry.

22-OHCR and CSA were synthetized as described (Supplementary Notes 1 and 2). Azide-BDP-FL (Jena Bioscience; cat. no. CLK-044-1) and Click-&-Go^™^ Cell Reaction Buffer Kit (Click Chemistry Tools; cat. no. 1263) were used for click chemistry labeling experiments. The plant material was incubated without or with CSA (20 μM) in liquid ½MS medium for 4 h, washed twice by PBS, fixed with 3.7% (v/v) formaldehyde in PBS for 15 min, washed twice with 3% (w/v) BSA in PBS. The samples were permeabilized by 0.5% (v/v) Triton^®^ X-100 in PBS for‘ 20 min at room temperature and washed three time with 3% (w/v) BSA in PBS. The click chemistry was done according to the manufacturer’s instructions (Click Chemistry Tools), using Azide-BDP-FL at a 4 μM concentration in the reaction mixture for 30 min. Samples were washed with 3% (w/v) BSA in PBS, counterstained by propidium iodide at 1:1000 dilution in H_2_O, and imaged.

### Aniline blue staining.

Seedlings were fixated and destained in 1:3 acetic acid/ethanol until the material was transparent (usually 2 h), then washed in 150 mM K_2_HPO_4_ for 30 min. Next, seedlings were incubated for at least 4 h in 150 mM K_2_HPO_4_ and 0.01% (w/v) aniline blue (staining solution) in tubes wrapped in aluminium foil for light protection, washed in 150 mM K_2_HPO_4_ for 5 min, and imaged.

### Callose immunostaining.

*Arabidopsis* seedlings were vertically grown on ½MS agar plates for 4 days, then transferred to fresh media with the following treatments for another 24 h: 200 nM BR, 1 μM BRZ, and DMSO mock. The immunolocalization procedure was done according to the published protocol^54^. In summary, seedlings were fixed in 4% (v/v) paraformaldehyde in microtubule stabilization buffer (MTSB; 50 mM PIPES, 5 mM EGTA, 5 mM MgSO_4_, pH 7 with KOH). Root tips were cut and mounted on poly-lysine-coated microscopy slides. Unspecific binding was prevented by blocking in neutral donkey serum before incubation with the antibody. The callose antibody (Australia Biosupplies) was diluted to 1:500 in MTSB containing 5% (v/v) neutral donkey serum and incubated with the samples for 4 h at room temperature. The secondary goat anti-Mouse IgG (H&L) - Alexa Fluor^™^ 594 was diluted to 1:500 in MTSB buffer containing 5% (v/v) neutral donkey serum and incubated with the samples for 1 h.

### RT–qPCR.

Total RNA was extracted by quantitative PCR with reverse transcription (RT–qPCR) from six-day-old seedlings with the RNeasy Mini Kit (Qiagen). Genomic DNA was eliminated by on-column digestion with RQ1 RNase-free DNase (Promega) during the isolation procedure. cDNA was generated from 1 μg of total RNA with qScript cDNA SuperMix (Quantabio) and analyzed on a LightCycler 480 II apparatus (Roche) with the SYBR Green I Master mix (Roche) according to the manufacturer’s instructions. Expression levels were normalized to those of ACTIN2. Primers are listed in Supplementary Table 2.

### Analysis of BL scRNA-seq.

To examine the transcriptional regulation of PD-related genes by BRs, we reanalyzed a previously described scRNA-seq dataset (GEO: GSE212230) in which wild type Arabidopsis plants were grown on 1μM BRZ for 7 days and transferred to 1 μM BRZ versus 100 nM BL for 2 hours^36^. First, we constructed a heatmap by calculating the log2 fold-change of BL 2 hours/BRZ using aggregated counts across all cell types and developmental stages. The heatmap was visualized with ComplexHeatmap (v2.10.0)^55^. Differentially expressed genes from each combination of cell type and developmental stage of the root^36^ were used to identify BL-regulated PD-related genes. We then plotted the log-normalized, ‘corrected’ counts produced by the SCTransform function^56^ for *PDLP3* and *CalS8/GSL4* on the two-dimensional uniform manifold approximation and projection (UMAP) embedding.

### Microscopy and image analysis.

Most of the images were captured by a Leica SP8X confocal microscope. Images were collected using Las-X software (v 3.5.0.18371). GFP and mCherry were excited at 488 nm and 594 nm and acquired at 500–530 nm and at 600-650 nm, respectively. For the study of the BR biosynthetic enzymes subcellular localization, images were taken by a X40/1.10 WATER objective and signal accumulation was used during confocal imaging for *Arabidopsis* lines related to ROT3 and DWF4. For the experiments related to the transgenic line *pBES1:gBES1-GFP, Arabidopsis* roots were mounted in propidium iodide (Sigma-Aldrich) (10 ng/ml) solution between slides and coverslips and images were taken by a X25/0.95 WATER objective. Nine cells from the root transition zone were used for BES1-GFP signal quantification. For the click chemistry experiments , the BDP-FL signal was detected with a 503 nm laser excitation and a 505-519 nm emission filter and images were taken by a X25/0.95 WATER objective. For *pDWF4:DWF4-GFP/pUBI10:PDLP1-mCherry* and *pROT3:ROT3-GFP/pUBI10:PDLP1-mCherry* in Extended Data Fig. 1g, roots were imaged under a vertical ZEISS LSM900 microscope equipped with a Plan-Apochromat M27 20×/0.8 n.a. objective. GFP and mCherry were excited at 488 nm and 587 nm and acquired at 410-546 nm and at 595-700 nm, respectively. For immunostaining, samples were imaged with a Zeiss LSM 880 microscope with a X40/NA 1.3 oil lens. Atto550 excitation was done with 0.3% of 561 nm power and fluorescence collected between 566 and 700 nm. ZEISS ZEN 3.3 (blue edition) software was used for image collection with Zeiss LSM 880 and Zeiss LSM 900 microscopes. Callose deposition at PD was quantified for whole root meristems with Fiji software (https://fiji.sc/) macroinstruction program (Supplementary Note 3). The callose signal at the cell plate was excluded from measurements. The experiment was repeated two times with similar results. For aniline blue staining, 405 nm laser excitation and a 505 nm long-pass emission filter was used for imaging. Image analysis was done in Fiji software. To measure the length of mature cortical cells, seedlings were stained with propidium iodide (Sigma-Aldrich) and images were taken with a X25/0.95 WATER objective cells. The cells from the root region, in which root hairs start to emerge (a sign of cell differentiation after cessation of elongation) were imaged and measured.

### Two-photon microscopy and cell diffusion assay with DRONPA-s.

Five-day-old 35S-DRONPA-s seedling were grown on ½MS plates under long-day conditions at 21 °C and 70 % humidity. Then, they were transferred to plates containing either DMSO or 200 nM BL for an additional 24 h. Before imaging, plants were incubated for 2 min in 1/500 propidium iodide (PI) in water solution. 15-20 roots were analyzed with 2-3 ROIs per root. For DRONPA-s assay, root tips were analyzed with a Leica TCS SP5-Multi photon confocal microscope set up. PI was excited at 488 nm and detected at 620-700 nm. Fluorescence intensities were quantified using Leica LAS AF software. DRONPA-s deactivation: Root tips were illuminated with 488 nm light for 45 sec at 70% of the total laser intensity (argon laser, 20 mW, Leica Microsystems). DRONPA-s activation: 3 ROIs (corresponding to 3 cells per root) were set to the center of the cell to avoid activation of the adjacent cells. ROIs were illuminated with 800 nm light for 5 sec using two –photon IR (Titane-Saphir pulsating laser). Laser power is circa 2,6 W with 20 % gain. DRONPA-s acquisition: detection was made 2 sec after single cell activation up to 120 sec, with 20 % 488 nm argon laser at emission 500-575 nm. DRONPA-s movement analysis: Mean fluorescence intensities were measured from the activated cell and the 2 adjacent cells in ImageJ. DRONPA-s movement was calculated as fluorescent signal moving from the activated cell into the adjacent cells. X-Y drifts were corrected with StackReg plugin. After normalization and background subtraction, values were transformed into percentage of the average fluorescence from the two adjacent cells. The activated cell is considered to be at 100% (e.g 100% of the DRONPA-s molecules were activated in the ROI). 15-20 roots were analyzed with 2-3 ROIs per root.

### PD index.

Plasmodesmata enrichment was assessed by calculating the fluorescence intensity of FP-tagged DWF4, PD localized MCTP3 and the ER protein HDEL^57^, at (i) plasmodesmata (indicated by PDLP1-mCherry or PDCB1-mCherry) and (ii) the cell periphery. Constructs of interest were transiently co-expressed in *N. benthamiana* leaves with PDLP1-mCherry or PDCB1-mCherry (plasmodesmata markers). Confocal images of leaf epidermal cells were acquired by sequential scanning of PDLP1-mCherry or PDCB1-mCherry in channel 1 and GFP/BFP-tagged proteins in channel 2. About twenty images of leaf epidermis cells were acquired for each combinations. The quantification was carried out according to standard protocol^25^. Individual images were processed using ImageJ by defining six regions of interest (ROI) at plasmodesmata (using plasmodesmata marker to define the ROI in channel 1) and twelve ROIs at the cell periphery outside plasmodesmata. The GFP/BFP-tagged protein mean intensity (channel 2) was measured for each ROI and then averaged for single image. The plasmodesmata index corresponds to intensity ratio between fluorescence intensity of protein of interest at plasmodesmata versus cell periphery outside of plasmodesmata. Roots expressing DWF4-GFP/ROT3-GFP and PDLP1-mCherry were imaged in similar fashion to tobacco leaves and PD index for more than 20 cell per transgenic lines (4 roots per line) were calculated as explained above.

### Statistical analysis.

All statistical analyses were carried out with GraphPad Prism v.8.0 and v.9.0 software. Two-tailed Student’s paired *t*-test was used for BES1 immunoblot analysis. Comparison of more than two genotypes or treatments was done with one-way analysis of variance (ANOVA), Tukey’s multiple comparison test was used in the comparison procedure. *** *P* < 0.001, ** *P* < 0.01, and * *P* < 0.05.

## Extended Data

## Supplementary Material

Supplementary data

Source Data Fig. 1 Statistical source data

Source Data Fig. 1 Unprocessed western blots

Source Data Fig. 2 Statistical source data

Source Data Fig. 2 Unprocessed western blots

Source Data Fig. 3 Statistical source data

Source Data Fig. 3 Unprocessed western blots

Source Data Fig. 4 Statistical source data

Source Data Extended Data Fig. 1 Statistical source data.

Source Data Extended Data Fig. 2 Statistical source data

Source Data Extended Data Fig. 2 Unprocessed western blots

Source Data Extended Data Fig. 3 Statistical source data

Source Data Extended Data Fig. 3 Statistical source data fig 3

Source Data Extended Data Fig. 4 Statistical source data

Source Data Extended Data Fig. 4 Unprocessed western blots

Source Data Extended Data Fig. 5 Statistical source data

## Data Availability

Numerical source data files and uncropped scans of blots are provided for figures and extended data figures. Primer lists, Fiji macro for callose deposition quantification and notes on synthesis of chemical compound used in this study can be found in Supplementary Information.
