# Supplementary material for: Plasmodesmata mediate cell-to-cell transport of brassinosteroid hormones
Source: Nat Chem Biol. Author manuscript; Available in PMC 2024 May 1. (PMC10729306; doi:10.1038/s41589-023-01346-x)
Supplement: Supplementary data [file NIHMS1948439-supplement-Supplementary_data.pdf]

nature chemical biology

Article

https://doi.org/10.1038/s41589-023-01346-x

Plasmodesmata mediate cell-to-cell transport of brassinosteroid hormones

In the format provided by the authors and unedited

Supplementary Information

Table of Contents:

Supplementary Table 1. PD-related DEGs from pseudobulk analysis of BL scRNA-seq.
Supplementary Table 2. Primers used in this study.
Supplementary Note 1. Synthesis of 22-hydroxycampesterol (22-OHCR): Synthetic procedure
Supplementary Note 2. Synthesis of castasterone-alkyne (CSA): Synthetic procedure
Supplementary Note 3. Macro used for quantification of callose deposition with Fiji software

Supplementary Table 1. PD-related DEGs from pseudobulk analysis of BL scRNA-seq.

gene	cell_type_dev_stage	BRZ.frq	BL.frq	logFC	p_adj.loc	contrast	up_dn_label	Name	type
AT2G33330	Elongation_Cortex	0.0219	0.137	3.78	1.00E-21	BL-BRZ	Up	PDLP3	callose deposition
AT2G33330	Transition Domain_Cortex	0.0444	0.175	2.77	6.41E-17	BL-BRZ	Up	PDLP3	callose deposition
AT4G03550	Elongation_Atrichoblast	0.184	0.478	1.67	1.14E-12	BL-BRZ	Up	CalS12/GSL5	callose deposition
AT4G03550	Transition Domain_Atrichoblast	0.643	0.786	0.996	2.09E-08	BL-BRZ	Up	CalS12/GSL5	callose deposition
AT3G14570	Transition Domain_Pericycle	0.0345	0.244	4.04	5.59E-08	BL-BRZ	Up	CalS8/GSL4	callose deposition
AT1G18650	Transition Domain_Cortex	0.0849	0.152	1.59	7.68E-07	BL-BRZ	Up	PDCB3	callose binding
AT1G05570	Proliferation Domain_Atrichoblast	0.0971	0.233	1.82	1.98E-06	BL-BRZ	Up	CalS1/GSL6	callose deposition
AT1G18650	Proliferation Domain_Atrichoblast	0.158	0.311	1.5	2.22E-06	BL-BRZ	Up	PDCB3	callose binding
AT5G43980	Elongation_Endodermis	0.239	0.12	-1.01	4.07E-05	BL-BRZ	Down	PDLP1	callose deposition
AT4G03550	Transition Domain_Trichoblast	0.298	0.472	0.792	8.13E-05	BL-BRZ	Up	CalS12/GSL5	callose deposition
AT2G33330	Proliferation Domain_Pericycle	0.0385	0.519	6.52	4.16E-04	BL-BRZ	Up	PDLP3	callose deposition
AT2G33330	Maturation_Pericycle	0.124	0.243	1.37	7.31E-04	BL-BRZ	Up	PDLP3	callose deposition
AT1G05570	Maturation_Atrichoblast	0.28	0.132	-1.26	0.00158	BL-BRZ	Down	CalS1/GSL6	callose deposition
AT4G03550	Elongation_Cortex	0.241	0.242	0.958	0.00174	BL-BRZ	Up	CalS12/GSL5	callose deposition
AT1G66250	Proximal Columella	0.0913	0.225	1.61	0.00241	BL-BRZ	Up	PdBG3	callose degradation
AT3G59100	Transition Domain_Pericycle	0.0345	0.221	2.96	0.00246	BL-BRZ	Up	CalS6/GSL11	callose deposition
AT1G70690	Distal Columella	0.154	0.0985	-0.876	0.00331	BL-BRZ	Down	PDLP5	callose deposition
AT1G05570	Proliferation Domain_Cortex	0.11	0.256	2.12	0.00342	BL-BRZ	Up	CalS1/GSL6	callose deposition
AT2G33330	Elongation_Pericycle	0.0848	0.238	1.45	0.00413	BL-BRZ	Up	PDLP3	callose deposition
AT1G70690	Elongation_Endodermis	0.147	0.189	0.621	0.0209	BL-BRZ	Up	PDLP5	callose deposition
AT3G14570	Elongation_Pericycle	0.0931	0.153	0.606	0.0288	BL-BRZ	Up	CalS8/GSL4	callose deposition
AT2G01630	Transition Domain_Pericycle	0.207	0.496	1.86	0.0323	BL-BRZ	Up	PdBG2	callose degradation
AT1G70690	Maturation_Atrichoblast	0.242	0.365	0.915	0.0435	BL-BRZ	Up	PDLP5	callose deposition
AT3G59100	Elongation_Pericycle	0.108	0.184	0.603	0.0475	BL-BRZ	Up	CalS6/GSL11	callose deposition

Supplementary Table 2. Primers used in this study.

Primer name	Primer sequence
Cloning primers	
C4H-FW	GGGGACAAGTTTGTACAAAAAAGCAGGCTTTATGGACCTCCTCTTGCTGG
C4H-RV	GGGGACCACTTTGTACAAGAAAGCTGGGTTACAGTTCCTTGGTTTCATAACG
PDLP1-FW	GGGGACAAGTTTGTACAAAAAAGCAGGCTTTATGAAACTCACCTATCAATT
PDLP1-RV	GGGGACCACTTTGTACAAGAAAGCTGGGTTATAAGCATCATATTTATTAC
PDLP5-FW	GGGGACAAGTTTGTACAAAAAAGCAGGCTTTATGATCAAGACAAAGACGAC
PDLP5-RV	GGGGACCACTTTGTACAAGAAAGCTGGGTTTTTACACCATTTCTCATCTGC
MCTP3-FW	GGGGACAAGTTTGTACAAAAAAGCAGGCTTTATGCAGAGACCACCTCCTGA
MCTP3-RV	GGGGACCACTTTGTACAAGAAAGCTGGGTTGAGCATGCAATCAGTTCTTGC
Genotyping primers	
DWF4-L	GGAAACAAAGAACAGACGATGATC
DWF4-R	GTTGCCATCTCCAAGGATTAAAG
CPD-L	AAGGTCCTACTTTATGCAGAA
CPD-R	AAAAACATAAGTGAGAAGGCCGAAT
CPD-ins	GCACGAGGGAGCTTCCA
LB1.3 for SALK mutants	ATTTTGCCGATTTCGGAAC
qPCR primers	
DWF4-FW-qpcr	TCCCTAGTGGGTGGAAAGTG
DWF4-RV-qpcr	CTGTTGCCATCTCCAAGGAT
PDLP5-FW-qpcr	ACCAGGATGCGTTGACCAAA

PDLP5-RV-qpcr	CTGAGCCACACCCTGAACAT
ACTIN2-FW	CCAGCAGATGTGGATCTCCAAG
ACTIN2-RV	TCCCATTCATAAAACCCCAGCTT

Supplementary Note 1

Synthesis of 22-hydroxycampesterol (22-OHCR): Synthetic procedure

p-Toluenesulfonic acid monohydrate (150 mg, 0.79 mmol) was added to a stirred solution of steroidal alcohol[1] (Scheme 1.1, compound **4**) (750 mg, 1.74 mmol) in 1,4-dioxane (40 mL) and water (10 mL) and the reaction mixture was heated at 75°C under argon for 2 h (Scheme 1). The mixture was allowed to cool down and quenched by addition of triethylamine (3 mL) and stirred for an additional 10 min. The reaction mixture was poured into water and extracted with ethyl acetate. The combined organic layers were dried over Na_2SO_4 and concentrated to dryness. The crude product was purified by column chromatography on silica gel (mobile phase 20% ethyl acetate in cyclohexane) to afford the 22-OHCR (Scheme 1.1, compound **1**) as a white solid (645 mg; 89% yield).

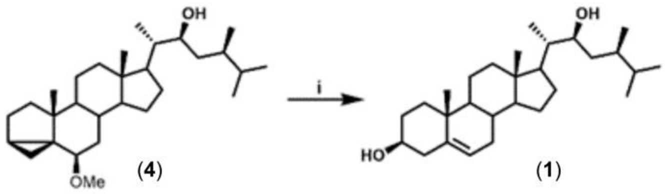

Scheme 1.1. Synthesis of 22-OHCR. i, *p*-TsOH/dioxane/H_2O, 75°C, 2 h.

The NMR spectra were taken on a JNM-ECA 500 spectrometer (JEOL, Tokyo, Japan; ^{1}H, 500 MHz; ^{13}C, 125 MHz) equipped with a 5 mm JEOL Royal probe. ^{1}H NMR and ^{13}C NMR chemical shifts (δ) were calibrated with tetramethylsilane (TMS, ^{1}H δ = 0 ppm) or $CDCl_3$ (^{1}H δ = 7.26 ppm, ^{13}C δ = 77.00 ppm). Chemical shifts are given in ppm (δ scale), coupling constants (J) in Hz. All values were obtained by first-order analysis. For ESI HRMS analysis, the samples were dissolved in methanol. Samples were analyzed with 1290 Infinity II Liquid Chromatographer (Agilent) with HPLC column infinityLab Poroshell 120 EC-C18 (4.6×50 mm; 2.7 µm). Mass accuracy of 1 ppm or less was achieved with the described instrumentation. Merck silica gel Kieselgel 60 (230–400 mesh) was used for column chromatography. Reagents and solvents were purchased (Sigma–Aldrich) and were not purified.

^{1}H NMR (500 MHz, $CDCl_3$ + 5% CD_3OD) δ 5.35 (m, ^{1}H, H-6), 3.77 (t, *J* = 6.9 Hz, ^{1}H, H-22), 3.52 (m, ^{1}H, H-3), 2.31–2.21 (m, ^{2}H), 2.02–1.90 (m, ^{3}H), 1.87–1.81 (m, ^{2}H), 1.01 (s, ^{3}H, CH_3), 0.89 (d,

J = 6.8 Hz, ^{3}H, CH_3), 0.87 (d, J = 7.0 Hz, ^{3}H, CH_3), 0.81 (d, *J* = 6.8 Hz, ^{3}H, CH_3), 0.83 (d, ^{3}H, *J* = 6.8 Hz, CH_3), 0.70 (s, ^{3}H, CH_3).

^{13}C NMR (126 MHz, $CDCl_3$) δ 140.70, 121.58, 71.57, 71.56, 56.61, 52.43, 49.99, 42.18, 42.07, 39.70, 39.26, 39.14, 37.16, 36.41, 35.20, 31.96, 31.85, 31.78, 31.43, 27.75, 24.15, 21.03, 19.92, 19.35, 17.76, 15.68, 11.73, 11.17.

HRMS (ESI+) calculated for $C_{28}H_{47}O$ ([(M-H_2O) + H]$^+$) 399.3627, found 399.3624.

Reference

1. Hurski, A. L., Ermolovich, Y. V., Zhabinskii, V. N. & Khripach, V. A. The development and use of a general route to brassinolide, its biosynthetic precursors, metabolites and analogues. *Org. Biomol. Chem.* **13**, 1446-1452 (2015).

Supplementary Note 2

Synthesis of castasterone-alkyne (CSA): Synthetic procedure

CSA (**2**) was synthesized in a two-step reaction with purification steps in-between, being clean-up on a normal phase silica gel column (Scheme 2.1). First, commercially available castasterone (**5**) was ligated to O-(carboxymethyl) hydroxylamine, providing the known compound castasterone-O-(carboxymethyl) oxime (CSCMO) (**6**). The carboxylic acid in CSCMO was then coupled to the primary amine functionality of propargylamine using a standard DMT-MM amide coupling (Scheme 2.1). The procedure followed was essentially the same as the one previously reported for Alexa Fluor 647-castasterone (AFCS)[1,2].

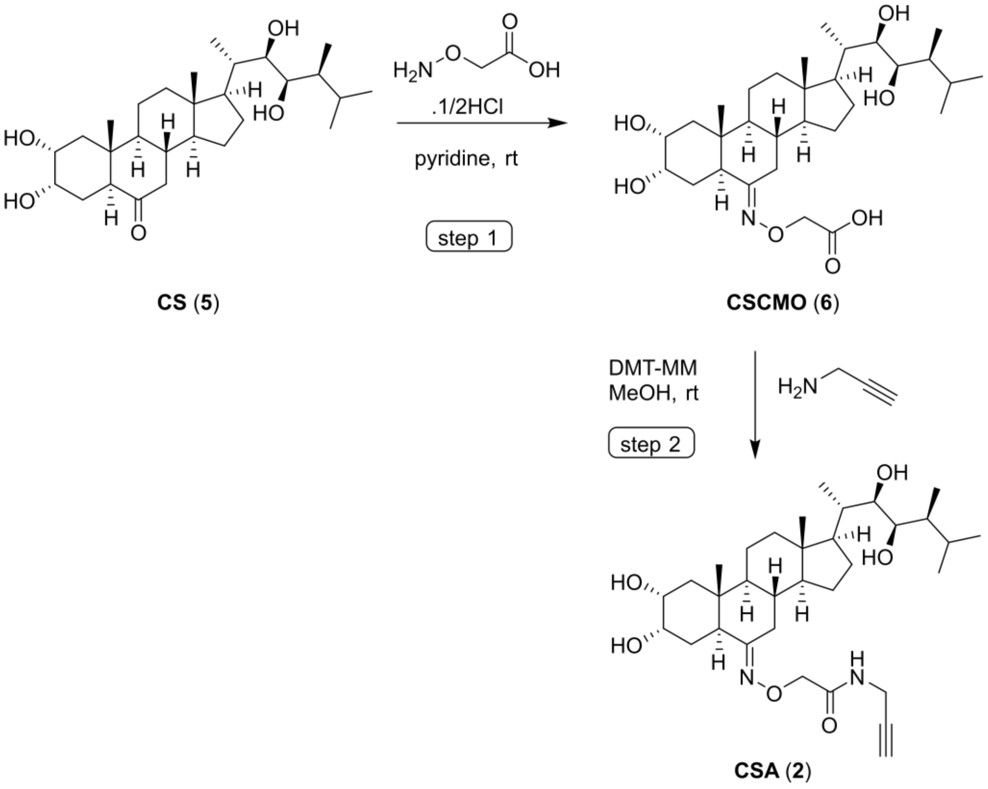

Scheme 2.1. Synthesis of CSA.

Chemicals and reagents

Castasterone (CS) (CAS, 80736-41-0) was kind gift form J. Oklestkova (Palacký University, Czech Republic), *O*-(carboxymethyl)hydroxylamine hemihydrochloride (CMHA) (CAS, 2921-14-4); 4-(4,6-dimethoxy-1,3,5-triazin-2-yl)-4-methylmorpholinium chloride (DMT-MM) (CAS, 3945-69-5); high-purity *N*,*N*-diisopropylethylamine (DIPEA) (CAS, 7087-68-5) (redistilled grade, >99%) and

3-amino-1-propyne, (propargylamine 98%) (CAS, 2450-71-7) were all purchased (Sigma-Aldrich) and used without further purification.

The solvents methanol, glacial acetic acid, pyridine, acetonitrile, and chloroform, were all purchased from Fisher Scientific at anhydrous high-performance liquid chromatography (HPLC) quality and used without further purification.

Chromatography material

Reactions were monitored by thin-layer chromatography (TLC), with SIL G25 UV254 TLC plates with 0.25 mm thick silica gel. The TLC plates were developed with an anisaldehyde stain solution (5% [v/v?] para-anisaldehyde and 1% [v/v?] sulfuric acid in ethanol), phosphomolybdic acid (PMA) (5% [v/v?] in ethanol) or ceric ammonium molybdate (CAM) (5% [v/v?] ammonium molybdate(VI).4H_2O (12054-85-2) + 1% [v/v?] cerium(IV)sulfate hydrate (17106-39-7) 10% [v/v?] sulfuric acid in water). Column chromatography is performed with ROCC N.V. silica gel (particle size from 0.060 to 0.200 mm), used without further treatments.

Analysis of obtained compounds

^{1}H Nuclear Magnetic Resonance (NMR) spectra were recorded with a resolution of 700, 400, or 300 MHz. The chemical shifts (δ) are expressed in ppm and the residual solvent peak is used as the internal standard ($CDCl_3$: ^{1}H = 7.26 ppm; ^{13}C = 77.00 ppm; $(CD_3)_2CO$: ^{1}H = 2.05 ppm; ^{13}C = 29.92 ppm). The multiplicity of the signals were designated by the following abbreviations: s, singlet; d, doublet; t, triplet; q, quadruplet; m, multiplet; br broadened; band, several overlapping signals.

Mass spectra (MS) were run with an Agilent ESI single quadrupole type detector VL. High resolution mass spectra (HRMS) are recorded on an Agilent Accurate-Mass Quadrupole Time-of-Flight mass spectrometer.

Step 1: synthesis of CSCMO

For the synthesis of CSCMO, the reported procedure cited above was followed exactly with 4.01 mg of castasterone (CS, 8.63 µmol) and 4.31 mg of *O*-(carboxymethyl)hydroxylamine hemichloride (CMHA, 39.6 µmol, 4.6 equivalents) in 0.3 mL of anhydrous pyridine. After overnight agitation, another 2.0 mg of CMHA was added and the reaction was heated to 60°C for 2 h to assure complete CS consumption. Volatiles were removed *in vacuo* and the residue was directly purified by means

of normal-phase column chromatography over silica gels. The column was eluted with a 20:1 mixture of chloroform and methanol, containing 0.5% (v/v?) glacial acetic acid, allowing obtention of the title compound in pure form after coevaporating three times with acetonitrile (approximately 4.8 mg; the maximum theoretical yield is 4.64 mg, i.e. near quantitative yield). The compound was used without further purification in the next step. This compound had a proton NMR spectrum identical to that reported previously.

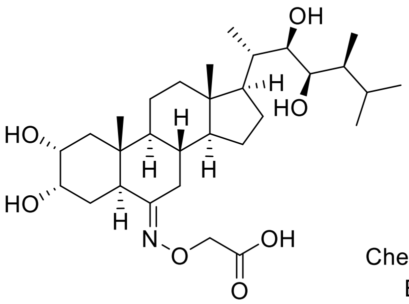

CSCMO

Chemical Formula: $C_{30}H_{51}NO_7$
Exact Mass: 537,36655
Molecular Weight: 537,73800

Step 2: synthesis of CSA

The castasterone-*O*-(carboxymethyl)oxime (CSCMO) obtained above (4.64 mg according to maximal theoretical yield, or 8.63 µmol) was redissolved in 0.464 mL of anhydrous methanol. From this stock solution, 0.200 mL (containing 2.00 mg CSCMO, 3.72 µmol) was transferred to a 5 mL round bottom flask already containing propargylamine (0.77 mg, 6.26 µmol, 1.68 equivalents), while under inert atmosphere. The resulting mixture was stirred at room temperature. A first portion of 4-(4,6-dimethoxy-1,3,5-triazin-2-yl)-4-methylmorpholinium chloride (DMT-MM) (~1.73 mg, 6.26 µmol, 1.68 equivalents) was added. After 10 min of stirring, a second portion of DMT-MM (~1.73 mg, 6.26 µmol, 1.68 equivalents) was added. After 30 min of additional stirring, a minor amount of CSCMO could still be detected via thin-layer chromatography (chloroform: methanol: acetic acid 85:15:1), so a third portion of DMT-MM (~1.73 mg, 6.26 µmol, 1.68 equivalents) was added. Upon complete consumption of CSCMO (after ~90 min total reaction time), the volatiles were removed *in vacuo* and the residue was directly purified via normal-phase column chromatography over silica gels. The column was eluted with a gradient of 4 to 10% anhydrous methanol in chloroform, allowing obtention of the title compound in pure form as judged by TLC, LC-MS, and 700 MHz NMR analysis, except for the presence of *N*-methyl-morpholine (10% by weight based on NMR integration). Approximately 2.6 mg of the compound was obtained (maximum theoretical yield of 2.14 mg), and was used in biological assays after thorough drying *in vacuo* to remove all *N*-methyl-morpholine that had coeluted to some degree with the compound.

Due to the dearth of material, extraction of all carbon resonance directly from the one-dimensional ^{13}C NMR spectrum proved difficult, but all other analyses were consistent with the expected product. The two-dimensional heteronuclear NMR (HSQC and HMBC) experiments provided the complete list of carbon resonances. The obtained data are fully consistent with the proposed structure. The spectra recorded on the sample included one-dimensional ^{1}H, tow-dimensional ^{1}H-{^{1}H} COSY, ^{1}H-{^{13}C} HSQC, ^{1}H-{^{13}C} HMBC, and ^{1}H-{^{1}H} NOESY. All carbon and proton resonances were found and assigned based on the observed two dimensional correlations. Conventional steroid numbering was used to indicate the castasterone carbons and protons (see formula). Chemical shifts for unresolved (overlapping) proton resonances are cited for the center of the observed two-dimensional correlation peaks.

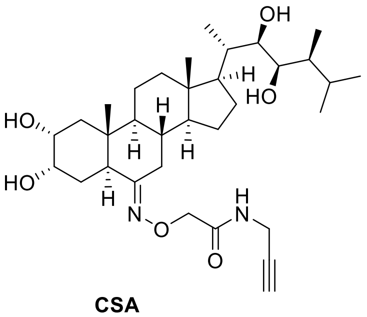

CSA

Chemical Formula: $C_{33}H_{54}N_2O_6$
Exact Mass: 574,39819
Molecular Weight: 574,80300

Compound characterization data

TLC (5% [v/v] methanol in chloroform): R_f= 0.23

TLC (15% [v/v] methanol in chloroform): R_f= 0.41

^{1}H NMR (chloroform-d, 700MHz**):** δ (ppm) = δ 6.36 [t(br), ^{1}H, J = 5.4 Hz, N*H*], 4.48 (s, ^{2}H, s, O-CH_2-CONH), 4.13 [ddd, ^{1}H, J = 17.4(AB), 5.4, 2.6 Hz, NH-C*H*H-C≡CH], 4.06 [ddd, ^{1}H, J = 17.4(AB), 5.4, 2.6 Hz, NH-C*H*H-C≡CH], 4.04 [s(br), ^{1}H, C3-H_{equat}], 3.77 [d(br), ^{1}H, J = 12.3 Hz, C2-H_{axial}], 3.72 [d(br), ^{1}H, J = C23-*H*], 3.56 [d(br), ^{1}H, J = 8.4 Hz, C22-*H*], 3.23 (dd, ^{1}H, J = 13.5, 4.5 Hz, C7-H_{equat}), 2.43 (dd, ^{1}H, J = 12.5, 2.5 Hz, C5-H_{axial}), 2.23 (t, ^{1}H, J = 2.6 Hz, CH_2-C≡C-*H*), 2.02-1.97 (m, ^{2}H, C12-H_{eqaut} and C15-*H*), 1.93 (ddd, ^{1}H, J = 15.3, 3.0, 3.0 (app. t) Hz, C4-H_{equat}), 1.76 (dd, ^{1}H, J = 12.5, 4.7 Hz, C1-H_{equat}), 1.75 (m, ^{1}H, C4-H_{axial}), 1.67 (m, ^{1}H, C16-*H*), 1.63 (m, ^{1}H, C25-*H*), 1.62 (m, ^{1}H, C11-*H*), 1.52 (m, ^{1}H, C8-*H*), 1.51 (m, ^{1}H, C20-*H*), 1.58 (m, ^{1}H, C17-H), 1.45 (m, ^{1}H, C1-H_{axial}), 1.32 (m, ^{1}H, C7-H_{axial}), 1.28 (m, ^{2}H, C11-*H* and C15-*H*), 1.26 (m, ^{1}H, C12-*H*), 1.25 (m, ^{1}H, C14-*H*), 1.22 (m, ^{1}H, C24-*H*), 1.17 (m, ^{1}H, C16-*H*), 1.11 [dd(br)d, ^{1}H, J = 11.5, 11.5 (app t), 2.8 Hz, C9-*H*], 0.97 (d, ^{3}H, J = 6.7 Hz, C26-H_3), 0.95 (d, ^{3}H, J = 6.7 Hz, C27-H_3), 0.91 (d,

^{3}H, J = 6.7 Hz, C21-H_3), 0.85 (d, ^{3}H, J = 6.9 Hz, C28-H_3), 0.75 (s, ^{3}H, C19-H_3), 0.69 (s, ^{3}H, C18-H_3). The four O*H* protons are not observed, and their methines are all broadened multiplets.

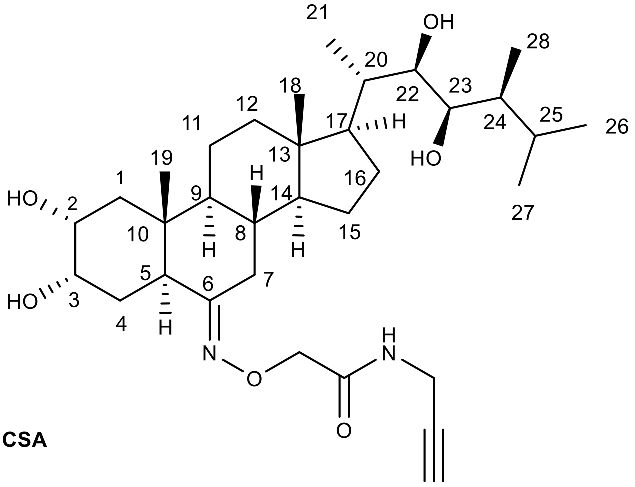

CSA

^{13}C NMR (chloroform-d, 175 MHz): δ (ppm) = 170.2 (amide, C_{quat}), 162.1 (oxime, C_{quat}), 79.0 (alkyne, CH_2-*C*≡CH, C_{quat}), 74.6 (C22, *C*HOH), 73.4 (C23, *C*HOH), 72.3 (O-*C*H_2-CONH), 71.6 (alkyne, C≡*C*-H), 68.4 (C3, *C*HOH), 68.3 (C4, *C*HOH), 56.4 (C14, *C*H), 53.9 (C9, *C*H), 52.2 (C17, *C*H), 43.2 (C5, *C*H), 42.4 (C13, C_{quat}), 40.1 (C10, C_{quat}), 40.0 (C24, *C*H), 39.8 (C1, *C*H_2), 39.4 (C12, *C*H_2), 36.7 (C20, *C*H), 35.6 (C8, *C*H), 30.8 (C25, *C*H), 30.3 (C7, *C*H_2), 28.6 (NH-*C*H_2-C≡*C*-H) 27.6 (C15, *C*H_2), 27.5 (C4, *C*H_2), 23.9 (C16, CH_2), 21.1 (C11, *C*H_2), 20.7 (C26, *C*H_3), 20.6 (C27, *C*H_3), 12.8 (C19, *C*H_3), 11.8 (C18, *C*H_3), 11.7 (C21, *C*H_3), 10.0 (C28, *C*H_3).

HSQC (chloroform-d, 700 MHz): 74.6×3.56, 73.4×3.72, 72.3×4.48, 71.6×2.24, 68.4×4.04, 68.3×3.77, 56.4×1.25, 53.9×1.11, 52.2×1.58, 43.2×2.43, 40.0×1.22, 39.8×(1.45 + 1.76), 39.4×(2.00 + 1.26), 36.7×1.51, 35.6×1.52, 30.8×1.63, 30.3×(3.23 + 1.32), 28.6×(4.06 + 4.12), 27.6×(2.00 + 1.28), 27.5×(1.75 + 1.92), 23.9×(1.67 + 1.17), 21.1×(1.28 + 1.62), 20.7×0.97, 20.6×0.95, 12.8×0.75, 11.8×0.69, 11.7×0.91, 10.0×0.85.

HMBC (chloroform-d, 700 MHz): 170.2×4.48, 162.1×(3.23 + 2.43 + 1.32), 79.0×(4.12 + 4.06 + 2.24), 74.6×(3.73 + 0.91), 73.4×(3.56 + 0.85), 56.4×(0.69 + 2.0), 53.9×(0.75 + 2.0 + 3.23 +), 52.2×(0.69 + 0.91) 43.2×(075 + 1.75 + 3.23), 42.4×(0.69 + 1.58 + 2.0), 40.1×(0.75 + 1.45 + 2.43), 40.0×(0.85 + 0.95 + 0.97 + 1.63), 39.8×0.75, 39.4×0.69, 36.7×0.91, 35.6×1.32, 30.8×(0.85 + 0.95 + 0.97 + 1.22 + 3.72), 28.6×2.24, 27.6×2.43, 23.9×1.25-1.27, 20.7×(1.63 + 0.95), 20.6×(1.63 + 0.97), 12.8×1.45, 11.8×1.58, 11.7×3.56, 10.0×(1.22 + 3.72).

NOESY (chloroform-d, 700 MHz): 6.36×4.48, 3.77×(1.75 + 0.75), 3.56×(1.58 + 0.85), 0.69×(1.17 + 1.28 + 1.52 + 2.00).

Formula: $C_{33}H_{54}N_2O_6$

MW (g/mol)**:** 574.8

HRMS (ESI) *m/z*: $[M + H]^+$ for $C_{33}H_{55}N_2O_6$: calculated 575.4055 vs 575.4056 obtained.

Copy of ^{1}H NMR of CSA in $CDCl_3$ (700 MHz), still containing ~10% unevaporated *N*-methyl-morpholine (also seen in LC-MS at *m/z* 102).

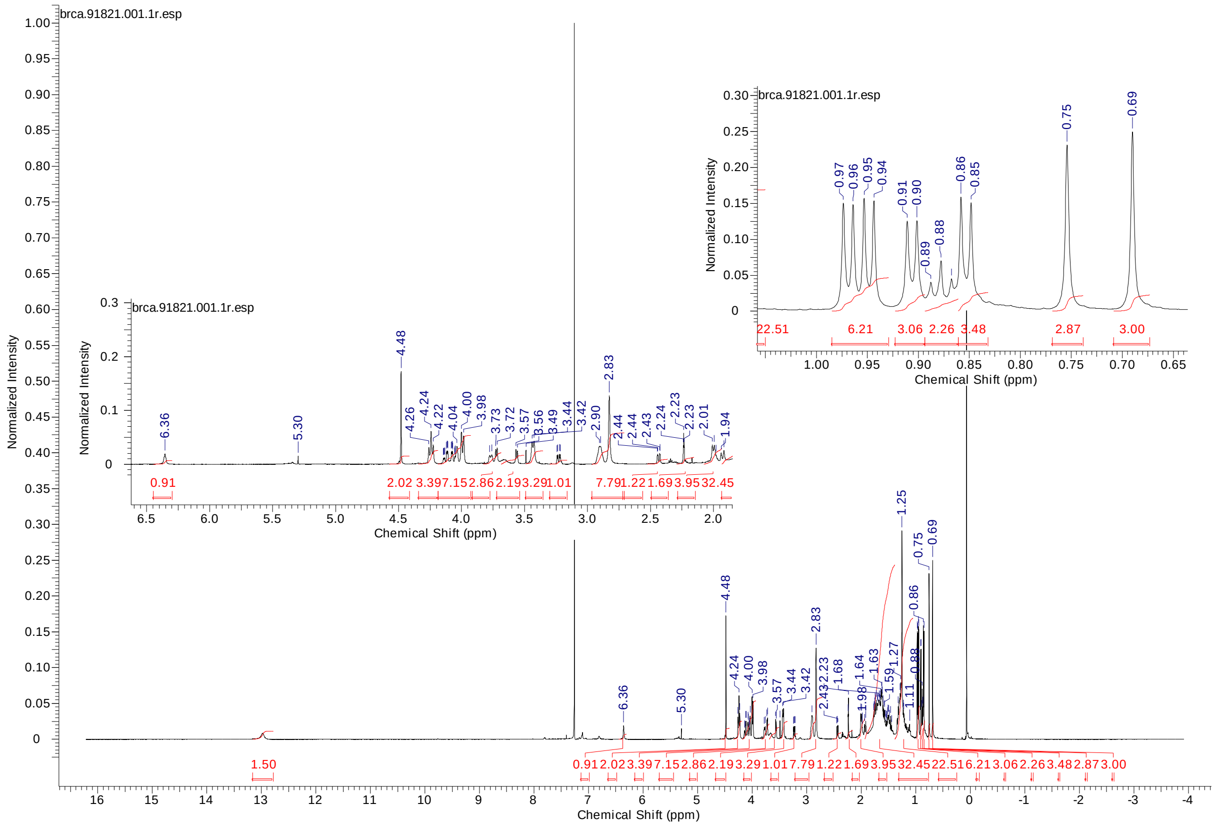

Resonances for *N*-methyl morpholine (protonated form):

13.0 [s(br), ^{1}H, N*H*], 4.24 [app. t(br), ^{2}H, J = ~12.3 Hz], 3.99 (d, ^{2}H, J = 11.8 Hz), 3.43 [d(br), ^{2}H, J = 10.8 Hz], 2.90 [t(br), ^{2}H, J = ~12 Hz, 2.83 [s(br), 3H, N-C*H*3]

Reference data reported previously[3]: (in DMSO-d6: 9.76 (s, ^{1}H), 3.94 (d, ^{2}H, J = 12.9 Hz), 3.61 (t, ^{2}H, J = 12.5 Hz), 3.34 (d, ^{2}H, J = 12.2 HZ), 3.03 (t, ^{2}H, J = 12.5 Hz), 2.79 (s, ^{3}H)

By proton integration, the weight percentage for the solvent contamination was approximately 10% in the measured sample.

Copy of ^{1}H-{^{13}C} HSQC NMR of CSA in $CDCl_3$ (700 MHz), still containing ~10% unevaporated *N*-methyl-morpholine (also seen in LC-MS at *m/z* 102).

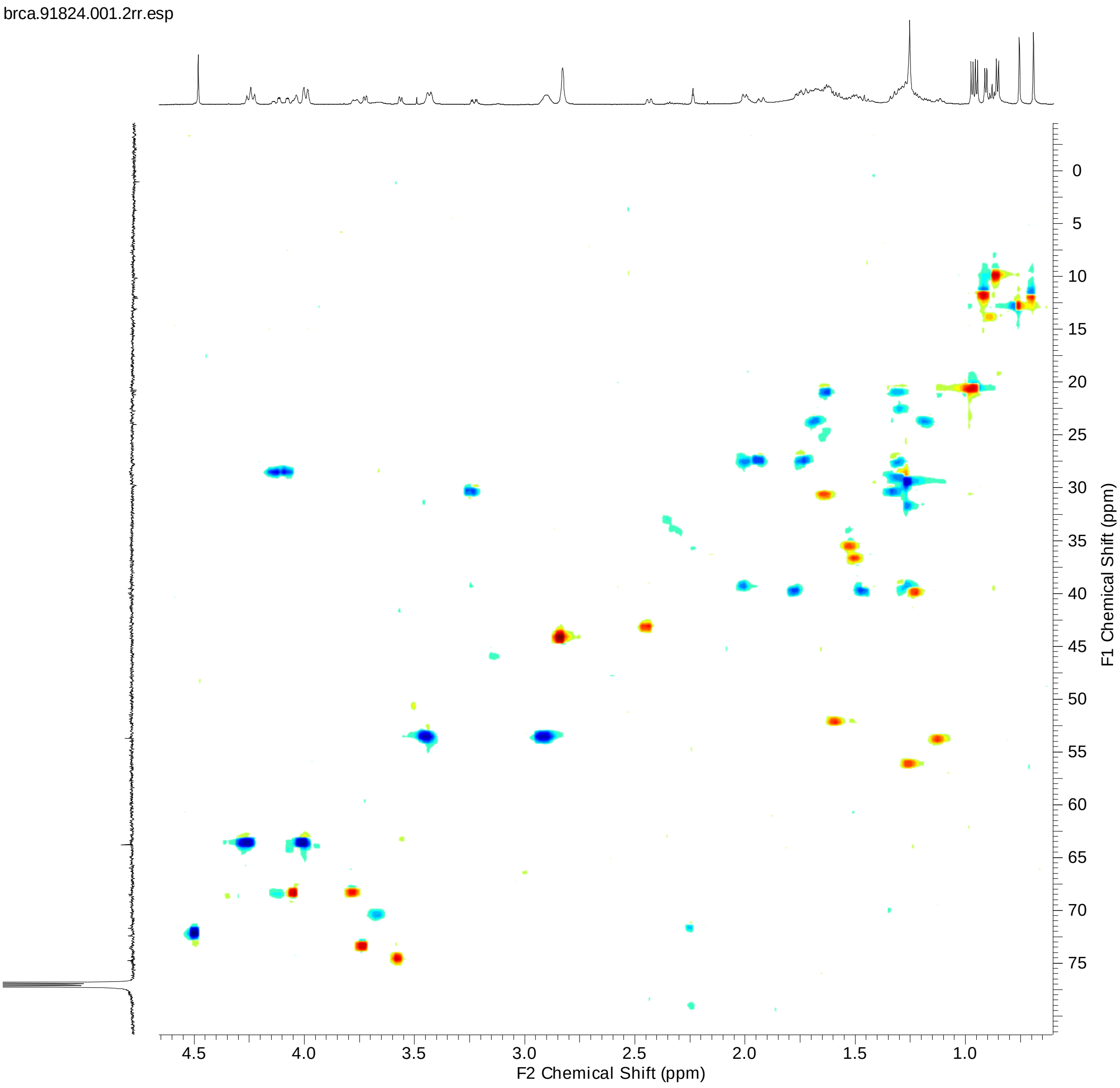

Copy of ¹H-{¹³C} HMBC NMR of CSA in $CDCl_3$ (700 MHz), still containing ~10% unevaporated *N*-methyl-morpholine (also seen in LC-MS at *m/z* 102).

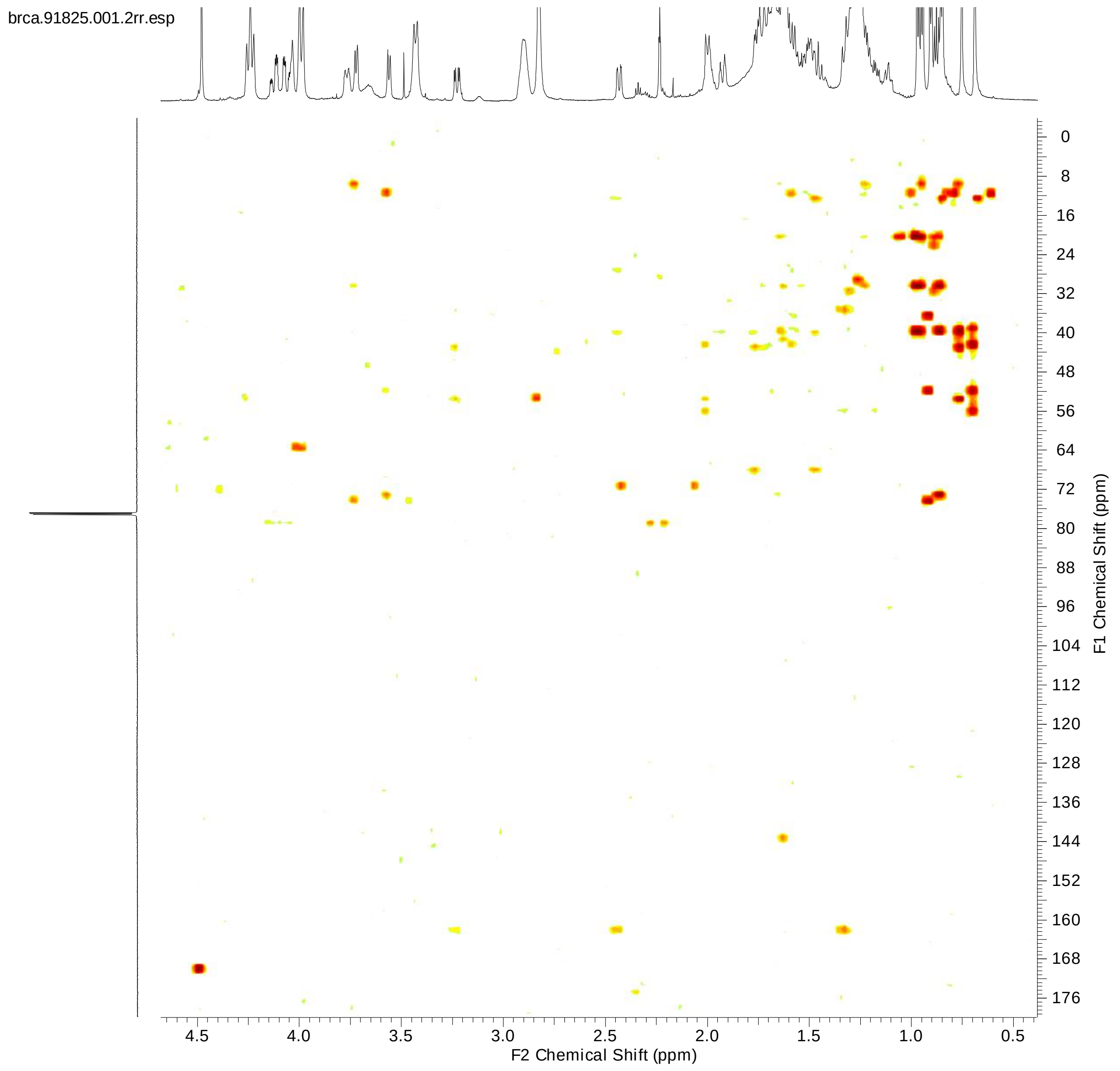

Copy of LC-MS analysis of compound after further drying *in vacuo*:

LCMS (ESI) retention time (*m/z*): 1.18 (*m/z* 102.1), 5.99 min (*m/z* 575.3)

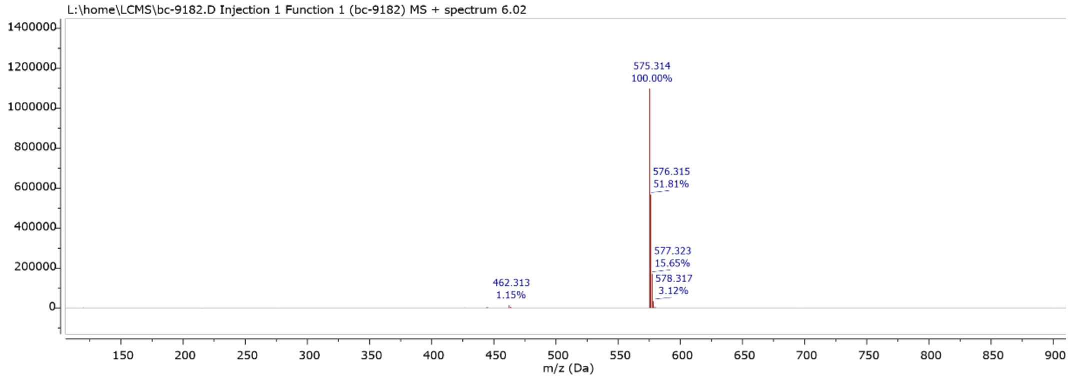

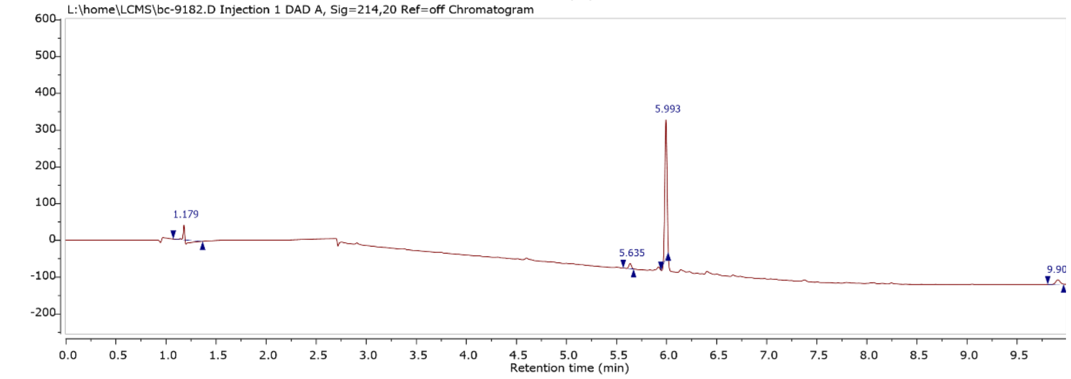

References

1. Winne, J. M., Irani, N. G., Van den Begin, J. & Madder, A. Synthetic protocol for AFCS: a biologically active fluorescent castasterone analog conjugated to an Alexa fluor 647 dye. Methods Mol. Biol. **1564**, 9-21 (2017).

2. Irani, N. G. et al. Fluorescent castasterone reveals BRI1 signaling from the plasma membrane. Nat. Chem. Biol. **8**, 583-589 (2012).

3. Tong, X., Ma, Y. & Li, Y. An efficient catalytic dehydration of fructose and sucrose to 5-hydroxymethylfurfural with protic ionic liquids. Carbohydr. Res. **345**, 1698-1701 (2010).

Supplementary Note 3

Macro used for quantification of callose deposition with Fiji software

```
title=getTitle();
path = getDirectory("image");
//separate the two channels, synchronize windows and apply the desired LUT to the callose channel
run("Split Channels");
run("Sync Windows");
run("Magenta Hot");
run("Enhance Contrast", "saturated=0.35");
run("Tile");
// Make a substack of the planes we want to include in the analysis and save it
waitForUser("Select first slice");
s1 = getSliceNumber();
waitForUser("Select last slice");
s2 = getSliceNumber();
run("Make Substack...", "  slices="+s1+"-"+s2);
saveAs("Tiff", path + title + "-C2-substack-" + s1 + "-" + s2 + ".tif");
titleC2substack = getTitle();
//Duplicate the image and create a background image to substrack from the original one
run("Duplicate...", "duplicate");
run("Gaussian Blur...", "sigma=60 stack");
titleC2subsBG = getTitle();
imageCalculator("Subtract create stack", titleC2substack,titleC2subsBG);
saveAs("Tiff", path + title + "-C2-substack-noBG" + s1 + "-" + s2 + ".tif");
titleC2subsnoBG = getTitle();
// Duplicate the image and perform some image-treatment to create a mask
selectWindow(titleC2subsnoBG);
run("Duplicate...", "duplicate");
run("Remove Outliers...", "radius=2 threshold=80 which=Bright stack");
run("Smooth", "stack");
run("Unsharp Mask...", "radius=6 mask=0.60 stack");
run("Remove Outliers...", "radius=4 threshold=80 which=Bright stack");
//Create the mask and save it
setThreshold(35, 255);
run("Convert to Mask", "method=Default background=Dark");
run("Options...", "iterations=2 count=1 do=Close stack");
saveAs("Tiff", path + title + "-C2-substack-" + s1 + "-" + s2 + "-Mask" + ".tif");
titleC2subsMask = getTitle();
//Use the mask to analyze particles referring to the original C2-substack
run("Set Measurements...", "area mean standard modal min integrated median display redirect=" +
titleC2substack + " decimal=3");
run("Analyze Particles...", "size=0.250-Infinity show=Masks display clear add in_situ stack");
selectWindow("Results");
saveAs("Results", path + title + "-C2-substack-" + s1 + "-" + s2 + "-results" + ".csv")
```

The obtained data files were treated in Excel. In each case, the regions of interest (ROIs) below and above a specific threshold were discarded, because they represent noise or large artefacts. The total integrated density of all ROIs in one root were summed up and presented in the graph grouped by genotype and treatment.
