# Supplementary material for: Plasmodesmata mediate cell-to-cell transport of brassinosteroid hormones
Source: Nat Chem Biol. Author manuscript; Available in PMC 2024 May 1. (PMC10729306; doi:10.1038/s41589-023-01346-x)
Supplement: Source Data Fig. 1 Unprocessed western blots [file NIHMS1948439-supplement-Source_Data_Fig__1__Unprocessed_western_blots.pdf]

Figure 1e

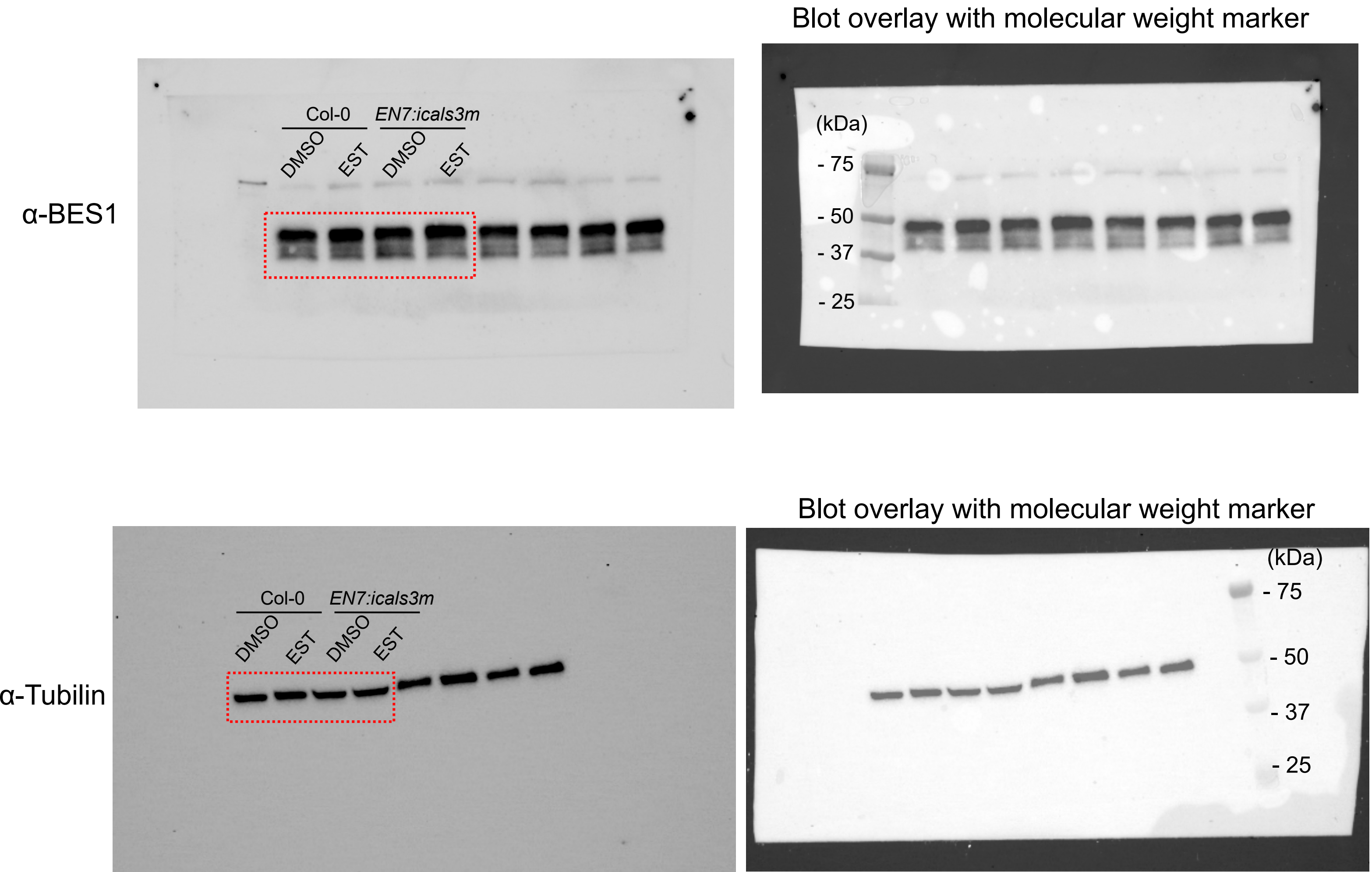

α-BES1
Col-0
EN7:icals3m
DMSO
EST
DMSO
EST
Blot overlay with molecular weight marker
(kDa)
- 75
- 50
- 37
- 25
α-Tubilin
Col-0
EN7:icals3m
DMSO
EST
DMSO
EST
Blot overlay with molecular weight marker
(kDa)
- 75
- 50
- 37
- 25
