# Supplementary material for: Plasmodesmata mediate cell-to-cell transport of brassinosteroid hormones
Source: Nat Chem Biol. Author manuscript; Available in PMC 2024 May 1. (PMC10729306; doi:10.1038/s41589-023-01346-x)
Supplement: Source Data Fig. 2 Unprocessed western blots [file NIHMS1948439-supplement-Source_Data_Fig__2__Unprocessed_western_blots.pdf]

Figure 2e

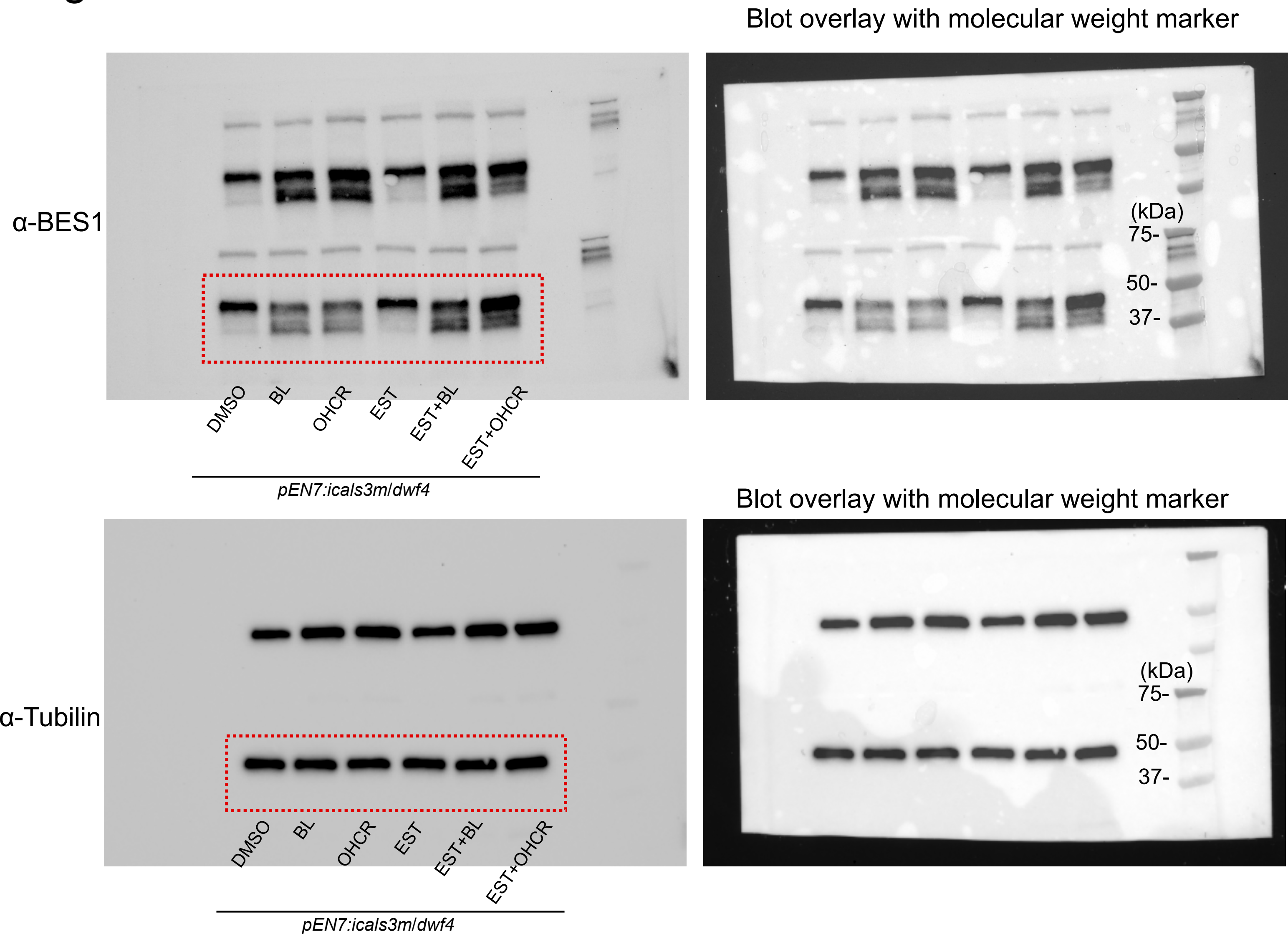
Blot overlay with molecular weight marker
α-BES1
DMSO
BL
OHCR
EST
EST+BL
EST+OHCR
pEN7:icals3m/dwf4
(kDa)
75-
50-
37-
Blot overlay with molecular weight marker
α-Tubilin
DMSO
BL
OHCR
EST
EST+BL
EST+OHCR
pEN7:icals3m/dwf4
(kDa)
75-
50-
37-
