# Supplementary figures and images for: Plasmodesmata mediate cell-to-cell transport of brassinosteroid hormones
Source: Nat Chem Biol. Author manuscript; Available in PMC 2024 May 1. (PMC10729306; doi:10.1038/s41589-023-01346-x)

Figure 3b

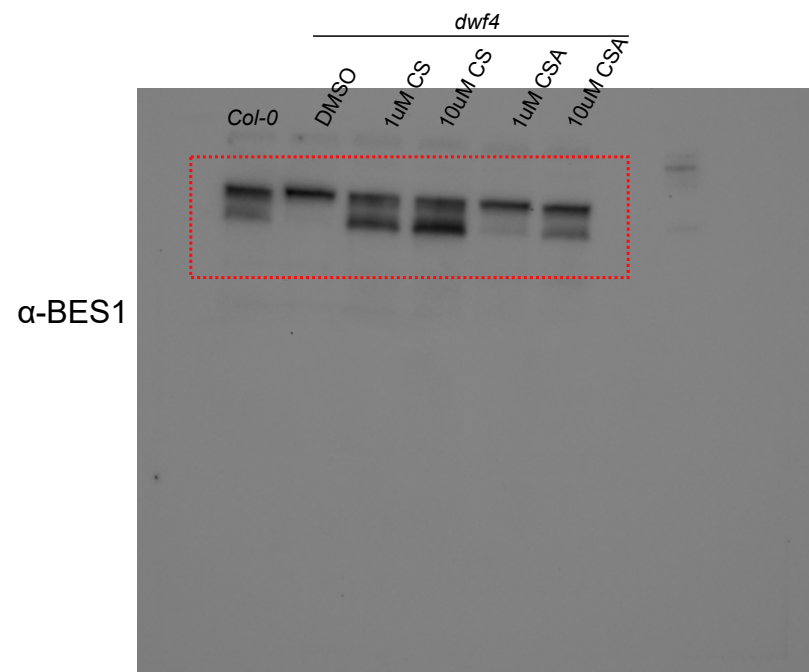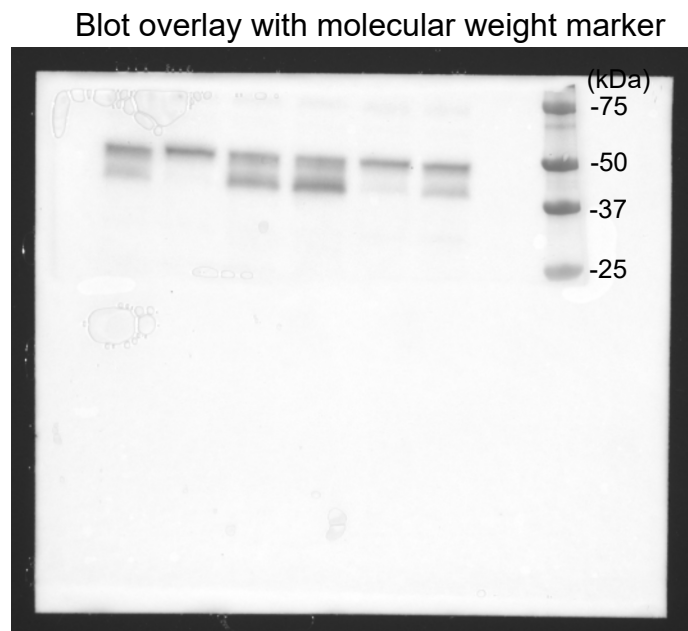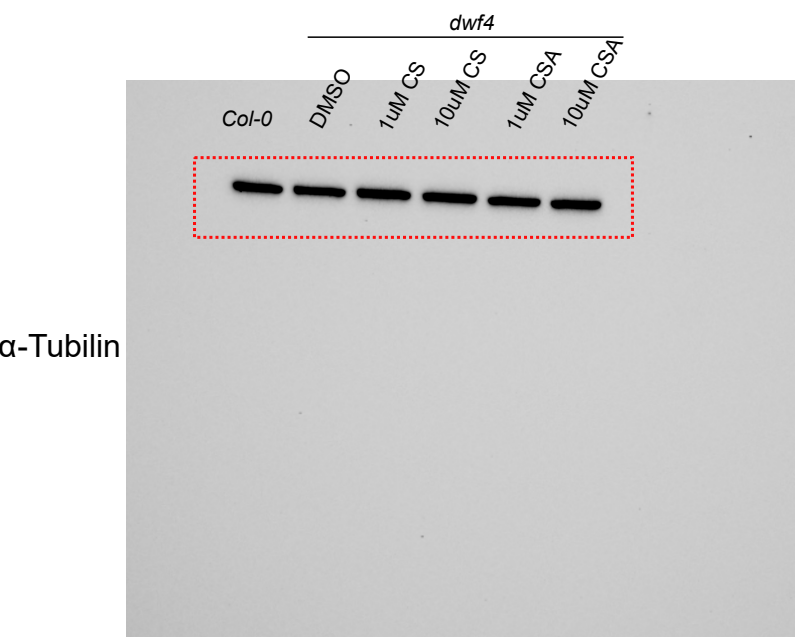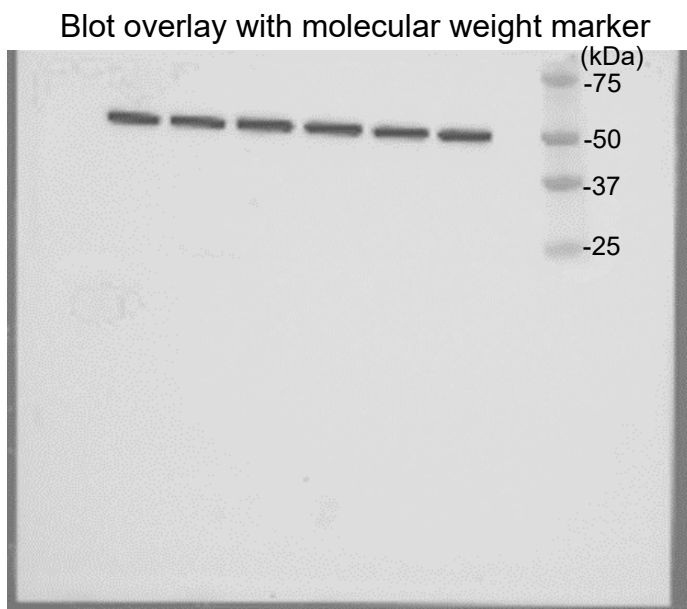

Supplement: Source Data Fig. 3 Unprocessed western blots [file NIHMS1948439-supplement-Source_Data_Fig__3__Unprocessed_western_blots.pdf]
