# Supplementary material for: Plasmodesmata mediate cell-to-cell transport of brassinosteroid hormones
Source: Nat Chem Biol. Author manuscript; Available in PMC 2024 May 1. (PMC10729306; doi:10.1038/s41589-023-01346-x)

Extended Data Fig. 2j

α-BES1

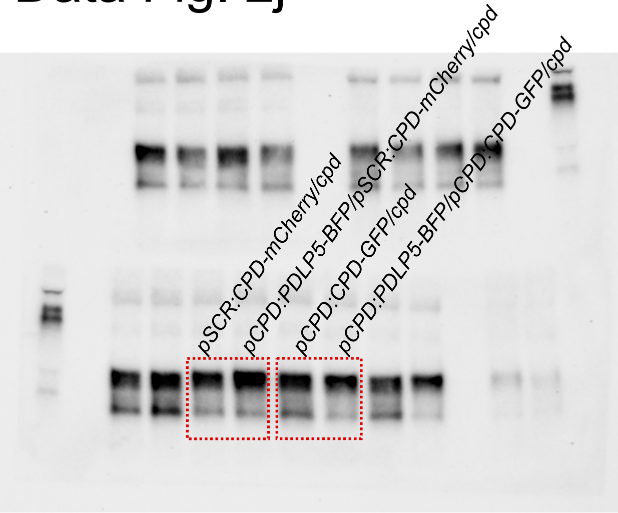

Blot overlay with molecular weight marker

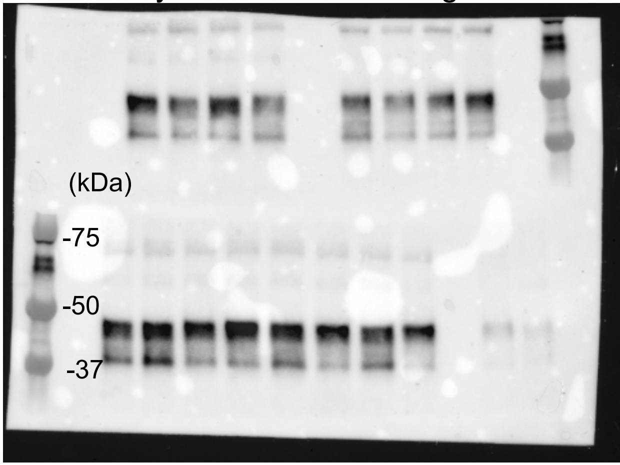

α-Tubilin

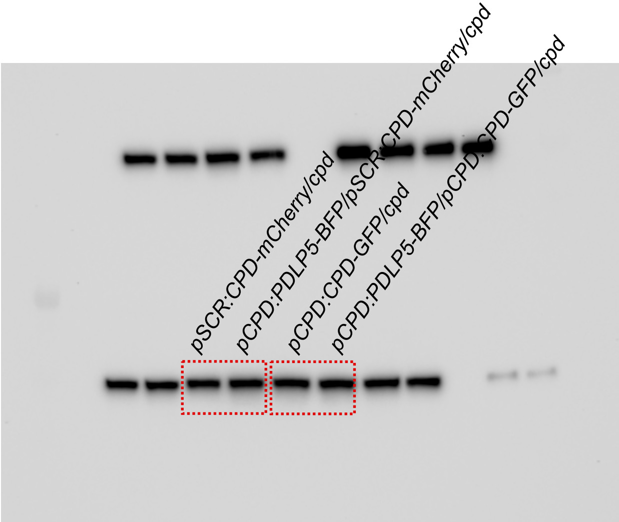

Blot overlay with molecular weight marker

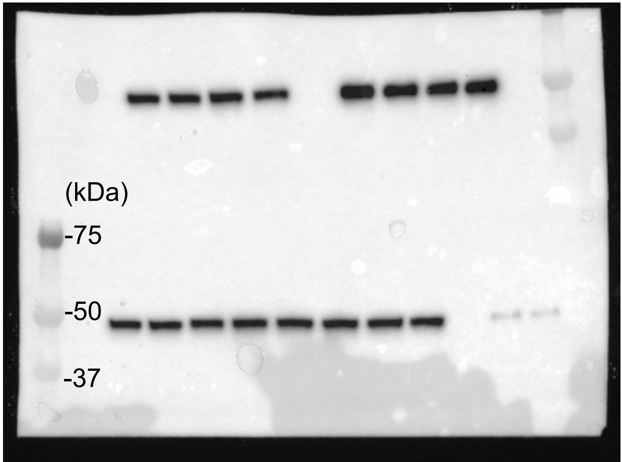

Supplement: Source Data Extended Data Fig. 2 Unprocessed western blots [file NIHMS1948439-supplement-Source_Data_Extended_Data_Fig__2__Unprocessed_western_blots.pdf]
